# CRISPR Gene Perturbations Provide Insights for Improving Bacterial Biofuel Tolerance

**DOI:** 10.3389/fbioe.2018.00122

**Published:** 2018-09-04

**Authors:** Peter B. Otoupal, Anushree Chatterjee

**Affiliations:** ^1^Department of Chemical and Biological Engineering, University of Colorado at Boulder, Boulder, CO, United States; ^2^BioFrontiers Institute, University of Colorado at Boulder, Boulder, CO, United States

**Keywords:** dCas9, gene expression, biofuels, tolerance, n-butanol, n-hexane, Pol1

## Abstract

Economically-viable biofuel production is often limited by low levels of microbial tolerance to high biofuel concentrations. Here we demonstrate the first application of deactivated CRISPR perturbations of gene expression to improve *Escherichia coli* biofuel tolerance. We construct a library of 31 unique CRISPR inhibitions and activations of gene expression in *E. coli* and explore their impacts on growth during 10 days of exposure to n-butanol and n-hexane. We show that perturbation of metabolism and membrane-related genes induces the greatest impacts on growth in n-butanol, as does perturbation of redox-related genes in n-hexanes. We identify uncharacterized genes *yjjZ* and *yehS* with strong potential for improving tolerance to both biofuels. Perturbations demonstrated significant temporal dependencies, suggesting that rationally designing time-sensitive gene circuits can optimize tolerance. We also introduce a sgRNA-specific hyper-mutator phenotype (~2,600-fold increase) into our perturbation strains using error-prone Pol1. We show that despite this change, strains exhibited similar growth phenotypes in n-butanol as before, demonstrating the robustness of CRISPR perturbations during prolonged use. Collectively, these results demonstrate the potential of CRISPR manipulation of gene expression for improving biofuel tolerance and provide constructive starting points for optimization of biofuel producing microorganisms.

## Introduction

Bacteria have long been investigated for their ability to produce renewable, biologically-derived replacements for petroleum-based fuels such as gasoline. Microbially produced biofuels have a promising future (Blazeck et al., 2014; Liu et al., 2015), with particular interest in straight-chain carbon alcohols (Trinh, 2012) and alkanes (Chen et al., 2013). Despite their potential, biofuels represent only ~2% of total transportation-based energy consumption (Jin et al., 2011), primarily due to their low economic competitiveness. This is limited to a large degree by the inherent toxicity such products exhibit to their hosts (Dunlop, 2011).

One particularly interesting biofuel is n-butanol due to its high energy density, low volatility, and ability to interface with our current gasoline-based infrastructure (Dürre, 2007; Qureshi and Ezeji, 2008). However, in a clear representation of the aforementioned tolerance issue, butanol is one of the most toxic biofuel compounds to microorganisms (Sardessai and Bhosle, 2002), with yields typically limited to a maximum of 2% vol/vol under optimal conditions (Knoshaug and Zhang, 2009; Xue et al., 2014). Engineering improved butanol tolerance is a key limiting factor to its economic viability and remains an elusive goal (Tian et al., 2013). Similar problems have plagued the progress of bringing other biofuels such as n-hexane to market (Liu et al., 2012).

Increasing microbial tolerance to biofuels would go a long way toward improving their economic competitiveness and remains a high-priority research goal. Many studies have explored improving the tolerance of specialized strains such as *Clostridium* (Tomas et al., 2004; Li et al., 2016; Wang S. et al., 2017) or *Synechocystis* (Anfelt et al., 2013; Kaczmarzyk et al., 2018). While attempts have been made to import heterologous biofuel pathways into the well-characterized and easy to use *Escherichia coli* (Atsumi et al., 2008; Nielsen et al., 2009; Zheng et al., 2009), the tolerance of this model organism still poses a significant barrier to exploring these pathways in *E. coli* to their full potential. In *E. coli*, n-butanol tolerance has been associated with oxidative stress response, respiration, transport, and metabolite synthesis (Rutherford et al., 2010; Reyes et al., 2013). While these studies have posed promising pathways to target, the extensive knowledge established in other strains has yet to be fully translated to *E. coli*. For instance, a 20–30% increase in membrane fluidity has been associated with n-butanol exposure in *Clostridium*, suggesting that membrane related genes could also be involved in improving *E. coli* n-butanol tolerance (Liu and Qureshi, 2009; Fletcher et al., 2016).

A promising approach to improving tolerance is to engineer alternative gene expression states. Manipulating gene expression is an essential metabolic engineering approach that has been previously applied to increase ethanol tolerance (Alper et al., 2006), and could similarly be applied to improving tolerance toward other biofuels (Erickson et al., 2014). This has a crucial advantage over gene knockout or insertion approaches in that it can be used to fine-tune biofuel pathways so as to not waste essential resources and restrict growth (Wang C. et al., 2017). Furthermore, manipulation of gene expression can be easily implemented into genetic feedback circuits for real-time pathway balancing during biofuel production (Jones et al., 2015). However, successful manipulation of transcriptional machinery to regulate specific genes has been difficult to achieve, preventing widespread implementation of such practices (Liu et al., 2014).

Utilizing CRISPR technology is a promising way to overcome these barriers. Deactivated versions of Cas9 have been developed to fine-tune expression patterns by inhibiting (Qi et al., 2013) or activating (Bikard et al., 2013) virtually any gene in a relatively facile manner. This has sparked renewed interest in engineering gene expression to enhance biofuel production (Hsu et al., 2014), as CRISPR-mediated gene modulation has the potential for fine-tuned optimization of cellular pathways (Deaner and Alper, 2017). Furthermore, while CRISPR-Cas9 has been applied toward the integration of heterologous genes (Li et al., 2015; Alonso-Gutierrez et al., 2017) increasing fatty acid production (Wu J. et al., 2017), improving butanediol production (Wu M. Y. et al., 2017), or redirecting metabolic flux (Wang C. et al., 2017), no work has explored the use of deactivated CRISPR systems for improving biofuel tolerance in *E. coli* (Cress et al., 2015b). Additionally, CRISPR interference has been used to improve *Klebsiella* n-butanol production 154%, demonstrating that there is similar potential for improving *E. coli* n-butanol tolerance (Wang M. et al., 2017).

**Figure 1:**
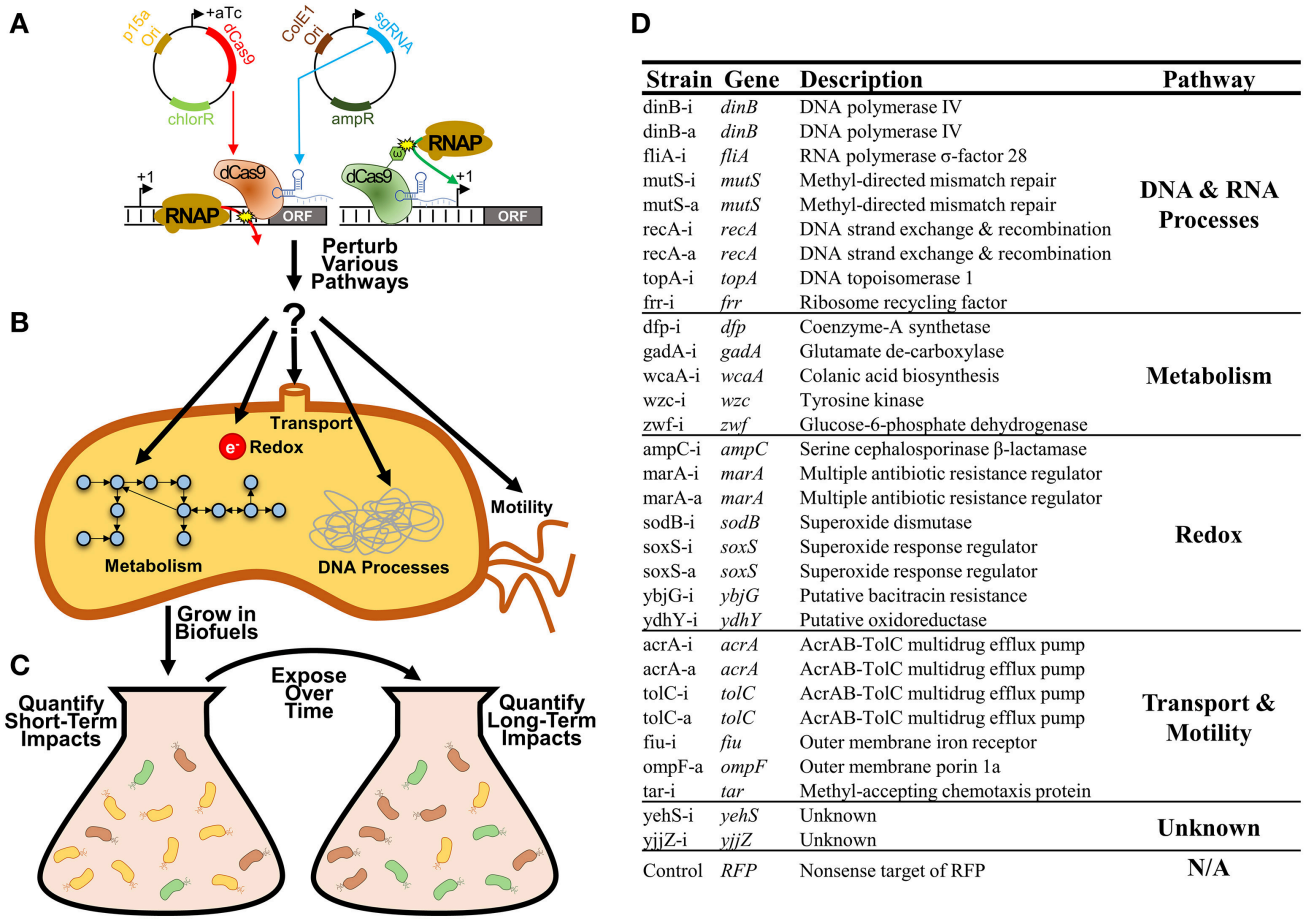
Improving bacterial tolerance to biofuels using CRISPR gene expression perturbation. **(A)** CRISPR perturbations of gene expression (both inhibition and activation) were designed for 31 *E. coli* genes and expressed using a two-plasmid system. **(B)** Strains used in this study. Whether CRISPR constructs were used to inhibit or activate gene expression are notated by -i or -a in the strain name respectively. **(C)** These perturbations were designed to disrupt expression of a variety of genes related to bacterial metabolism, redox, transport, various DNA and RNA processes, and motility. **(D)** Strains harboring these individual perturbations were exposed to biofuels (n-butanol and n-hexane) over multiple days, with the characterization of both short-term and long-term growth impacts.

Here we systematically explore the growth impacts of a library of 31 CRISPR inhibitions or activations of *E. coli* gene expression during exposure to either n-butanol or n-hexane (Figure 1A). These CRISPR constructs were targeted to genes involved in a broad range of cellular processes including metabolism, redox, transport, DNA, and RNA processes, and motility, as all have been implicated for their importance in determining biofuel tolerance capacity (Sardessai and Bhosle, 2002; Dunlop, 2011; Erickson et al., 2014, 2017; Otoupal et al., 2017; Figures 1B,C). We explored both inhibition and activation of gene expression, as both approaches could feasibly lead to optimization of tolerance. As growth phenotypes can be time-sensitive, we explored growth impacts over 10 days of exposure to identify perturbations that impact growth phenotypes in either the short-term (1 day) or long-term (10 days), as each result points to different approaches that could be implemented (Figure 1D).

Our CRISPR perturbation approach reveals a number of promising gene targets whose expression could be engineered for improved biofuel tolerance. Manipulation of metabolism-related genes, as well as membrane and periplasm related genes, appears the most promising pathways for increasing tolerance to n-butanol. Conversely, redox genes appear to be more influential in improving n-hexane tolerance. Strong temporal effects were identified under both conditions, suggesting that time-sensitive alterations of gene expression should be taken into consideration while engineering improved biofuel tolerance. We also present evidence that these perturbations are stable by artificially introducing a hyper-mutator phenotype (increasing basal mutation rates ~2,600-fold) during exposure to n-butanol (Camps et al., 2003; Alexander et al., 2014). Despite this increased mutation rate, perturbations largely demonstrate the same relative impact on growth phenotypes as before, suggesting that CRISPR perturbations maintain efficacy over prolonged periods. Together, these results demonstrate the power of CRISPR perturbations for improving biofuel tolerance.

## Materials and methods

### CRISPR plasmid and strain construction

Addgene plasmids #44249 and #44251 were used for expression of dCas9 and sgRNA respectively (obtained directly from Addgene). These plasmids harbor the chloramphenicol (cm) and ampicillin (amp) resistance markers respectively. Native 44251 targets the ORF of RFP, which is not present in any of the strains used in this study and was therefore used as the nonsense control sgRNA target sequence. Plasmid pPO-dCas9ω was constructed in a previous study (Otoupal et al., 2017) and used for expression of dCas9-ω alongside 44251. Unique sgRNA targets were constructed by PCR amplifying cloning inserts (primers obtained from Integrated DNA Technologies) replacing the RFP target sequence with the new target sequence for each gene. Inserts were flanked with SpeI and ApaI restriction sites. Plasmid 44251 digested with SpeI and ApaI (New England Biolabs) was used as the cloning backbone. Digested inserts were gel extracted (GeneJET Gel Extraction Kit, Thermo Fisher Scientific) and ligated (T4 DNA ligase, New England Biolabs) alongside this backbone and transformed into electrocompetent NEB 10-β (New England Biolabs). Final constructs were recovered using Zyppy Plasmid Miniprep Kit (Zymo Research Corporation) and confirmed by sequencing (via GENEWIZ) before transformation into chemically competent *E. coli* MG1655 (ATCC 700926) harboring either dCas9 or dCas9-ω plasmids for gene repression or activation respectively. Exact gene targets for each sgRNA are listed in Supplementary Table S1. The successful perturbation of gene expression using this CRISPR system was confirmed using quantitative real-time PCR in previous studies (Erickson et al., 2015, 2017; Otoupal et al., 2017). These constructs were built to perturb gene expression roughly 10-fold from basal levels.

### Error-prone strain construction

Strain JS200 expressing temperature-sensitive *polA* was obtained from Addgene (#11722) harboring the pEP Pol1 plasmid (error-prone *polA* D424A, I709N, A759R with reduced fidelity) with cm resistance marker. The plasmid was miniprepped from the strain, after which the strain's plasmid was removed by growing for 5 days at 30°C in 3 mL LB cultures, with 1:1,000 dilution into fresh culture every 24 h. The culture was streaked on plain LB agar plates at the end of this exposure period to obtain individual colonies. These colonies were screened for successful plasmid removal by plating in both the presence and absence of cm. A colony that grew only in the absence of cm was picked and saved to obtain strain JS200 with no plasmid.

Plasmids dCas9 and dCas9-ω were PCR amplified as Gibson Assembly inserts, while plasmid pEP Pol1 was PCR amplified as a Gibson Assembly backbone. Primers are listed in Supplementary Table S2 (obtained from Integrated DNA Technologies, Supporting Information). Successful PCR products were gel extracted, and Gibson Assembly was performed to insert pEP into dCas9 and dCa9-ω plasmids. A home-made Gibson Assembly mix was prepared using 2 μL Taq DNA Ligase (New England Biolabs M0208S), 0.25 μL Phusion High-Fidelity DNA polymerase (New England Biolabs, M0530S), 0.008 μL T5 exonuclease (New England Biolabs, M0363S), and 4 μL home-made ISO buffer. Gibson controls using only insert or backbone were run in parallel to confirm successful assembly. Constructs were transformed into electrocompetent NEB10-β, plasmids were recovered and run on a gel to confirm appropriate sizes, and submitted for sequencing confirmation. Plasmids were then transformed into empty chemically competent JS200, with overnight growth at 30°C with 35 μg/mL cm selection. Successful transformants of Pol1-dCas9 and Pol1-dCas9-ω were picked and grown overnight at 30°C. Each strain was made chemically competent (Zymo Mix & Go! Transformation Kit) and immediately transformed with each of the individual sgRNA targets, with growth at 30°C. To prevent excessive mutation before the start of the experiment, transformation plates were used directly to inoculate 4 biological replicates grown overnight at 30°C for the experiment represented in Figure 7. Experiments using these strains included 100 μM IPTG to drive expression of error-prone Pol1.

### Growth and media conditions

All cultures were grown in Lennox Luria-Bertani Broth (LB) (Sigma-Aldrich). Media was supplemented with amp (100 μg/mL, Sigma-Aldrich) to maintain a selection of sgRNA plasmids, or supplemented with cm (35 μg/mL, Sigma-Aldrich) to maintain a selection of dCas9/dCas9-ω/pEP Pol1 plasmids. Unless noted, amp and cm were always included in media. Growth of gene knockout strains was performed without supplementation of any antibiotic. Expression of dCas9/dCas9-ω during experiments was driven by supplementation of 50 ng/mL anhydrotetracycline (aTc, Sigma-Aldrich). Expression of error-prone Pol1 during experiments was driven by supplementation of 100 μM Isopropyl-β-D-thiogalactosidase (IPTG, Sigma-Aldrich). All cultures were grown at 37°C, with shaking at 225 rpm unless otherwise noted. Growth at 30°C was used during cloning of the error-prone strains in order to drive expression of wild-type Pol1.

### Growth assays during biofuel exposure

**Figure 2:**
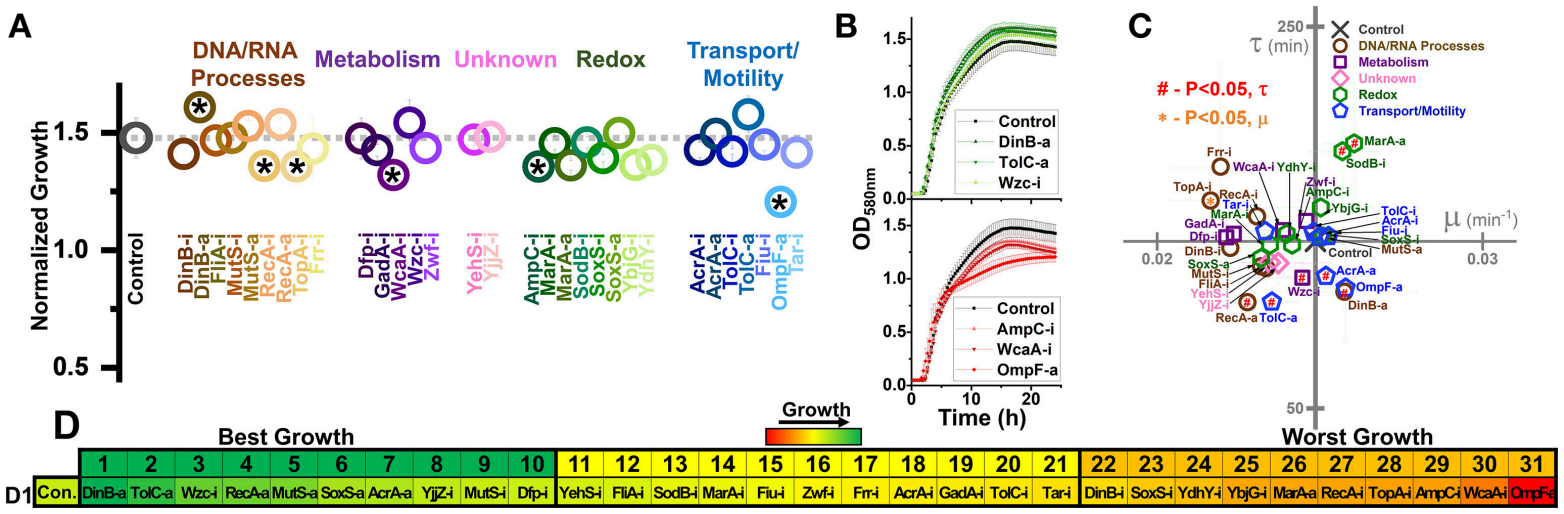
Growth of *E. coli* harboring CRISPR gene perturbations in the absence of biofuels. **(A)** Normalized growth (maximum OD/starting OD) of all strains. Strains are organized based on pathways affected by perturbation, and a dashed line extends from the control for comparison. Asterisks indicate significant differences in relation to the control (*P* < 0.05). A two-tailed type II *t*-test was used to calculate significance relative to the control. **(B)** Growth curves of the three strains growing to the highest levels (green, top) or lowest levels (red, bottom). **(C)** Growth rates (μ) and lag times (τ) of each strain in relation to the control strain, located at the intersection of the x- and y-axes. Pathways of the affected perturbation are again indicated using symbol and color. Red #significant differences in lag times, while orange *Significant differences in growth rates, relative to the control. **(D)** Organized rankings of strains by highest growth reached, with the color scale to indicate relative growth. The top 10 and bottom 10 are indicated as “best growth” and “worst growth,” respectively. All error bars represent the standard deviation of four biological replicates.

**Figure 3:**
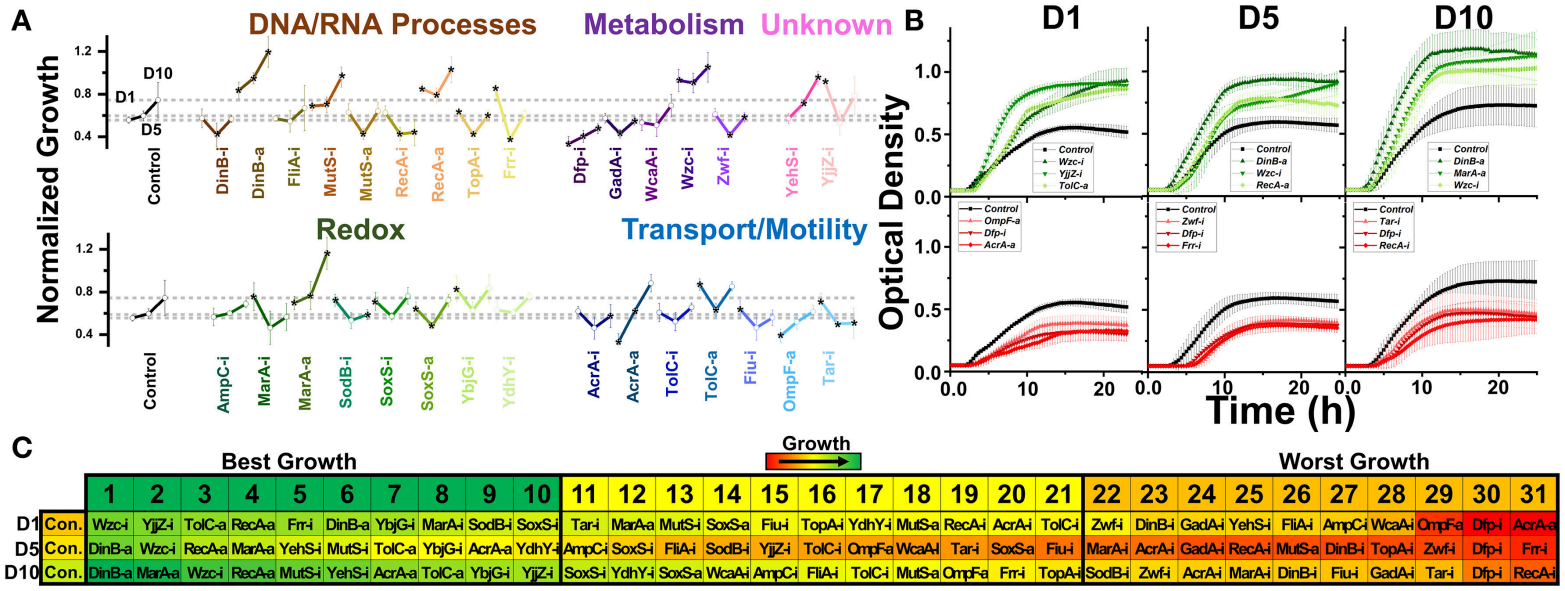
Normalized growth (maximum OD/starting OD) of *E. coli* harboring CRISPR gene perturbations during 0.5% vol/vol n-butanol exposure. **(A)** Change in growth of each strain over 10 days of exposure, with quantification on days one (D1), five (D5), and ten (D10). Strains are organized based on pathways affected by perturbation. Dashed lines extend from the control for each experimental day. A two-tailed type II *t*-test was used to calculate significance (as indicated by *P < 0.05) relative to the control on the same experimental day. **(B)** Growth curves of the three strains growing to the highest levels (green, top) or lowest levels (red, bottom) on D1, D5, and D10. **(C)** Organized rankings of strains with highest growth reached on each day, with the color scale to indicate relative growth. The top ten and bottom ten are indicated as “best growth” and “worst growth,” respectively. All error bars represent the standard deviation of eight biological replicates.

**Figure 4:**
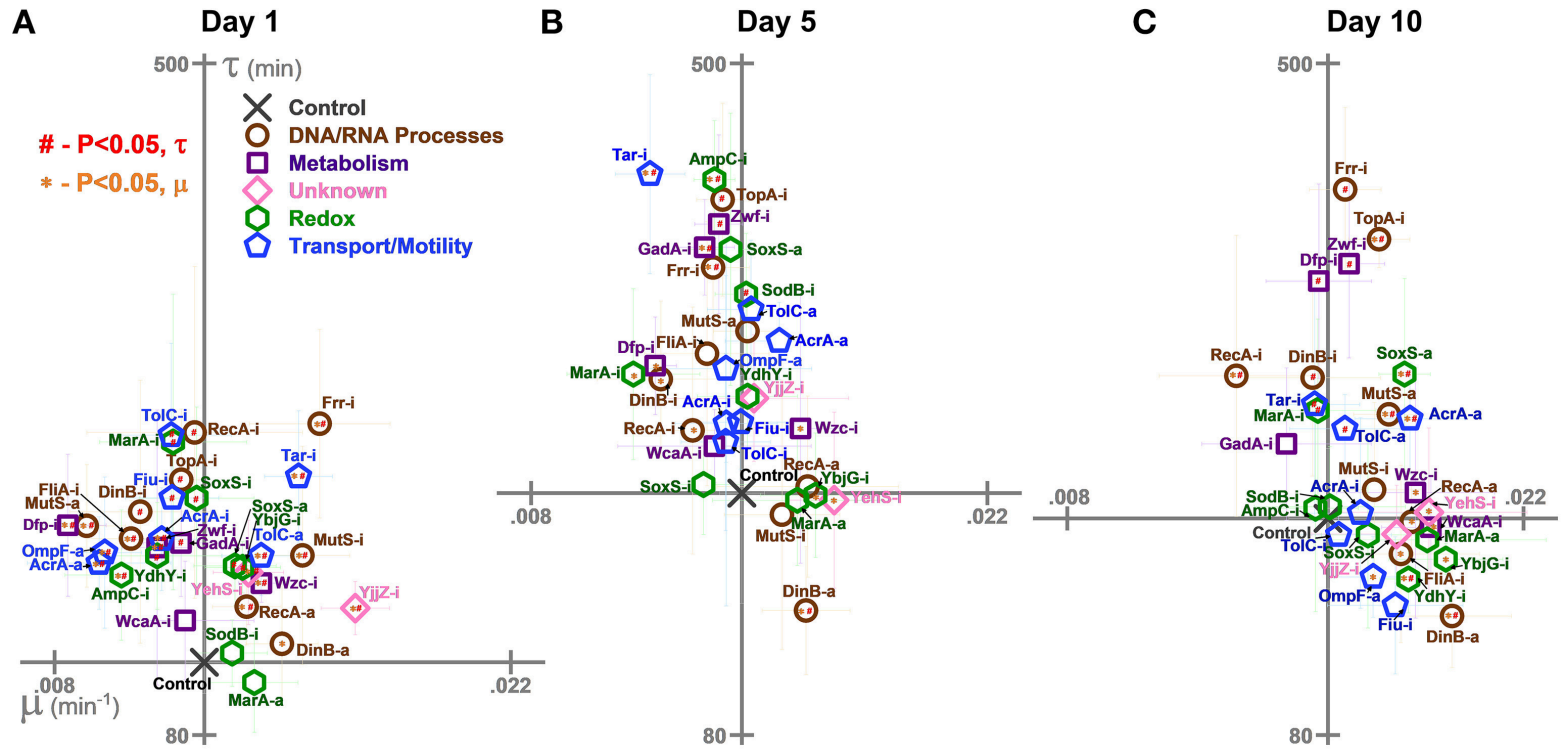
Growth rates (μ) and lag times (τ) of *E. coli* harboring CRISPR gene perturbations during 0.5% vol/vol n-butanol exposure. These growth characteristics were quantified on **(A)** day one, **(B)** day five, **(C)** and day 10 of the experiment. Scales are set to intersect the control in each graph. A two-tailed type II *t*-test was used to calculate significance (*P* < 0.05) relative to the control in growth rates (orange *) and lag times (red #). Error bars represent the standard deviation of eight biological replicates.

**Figure 5:**
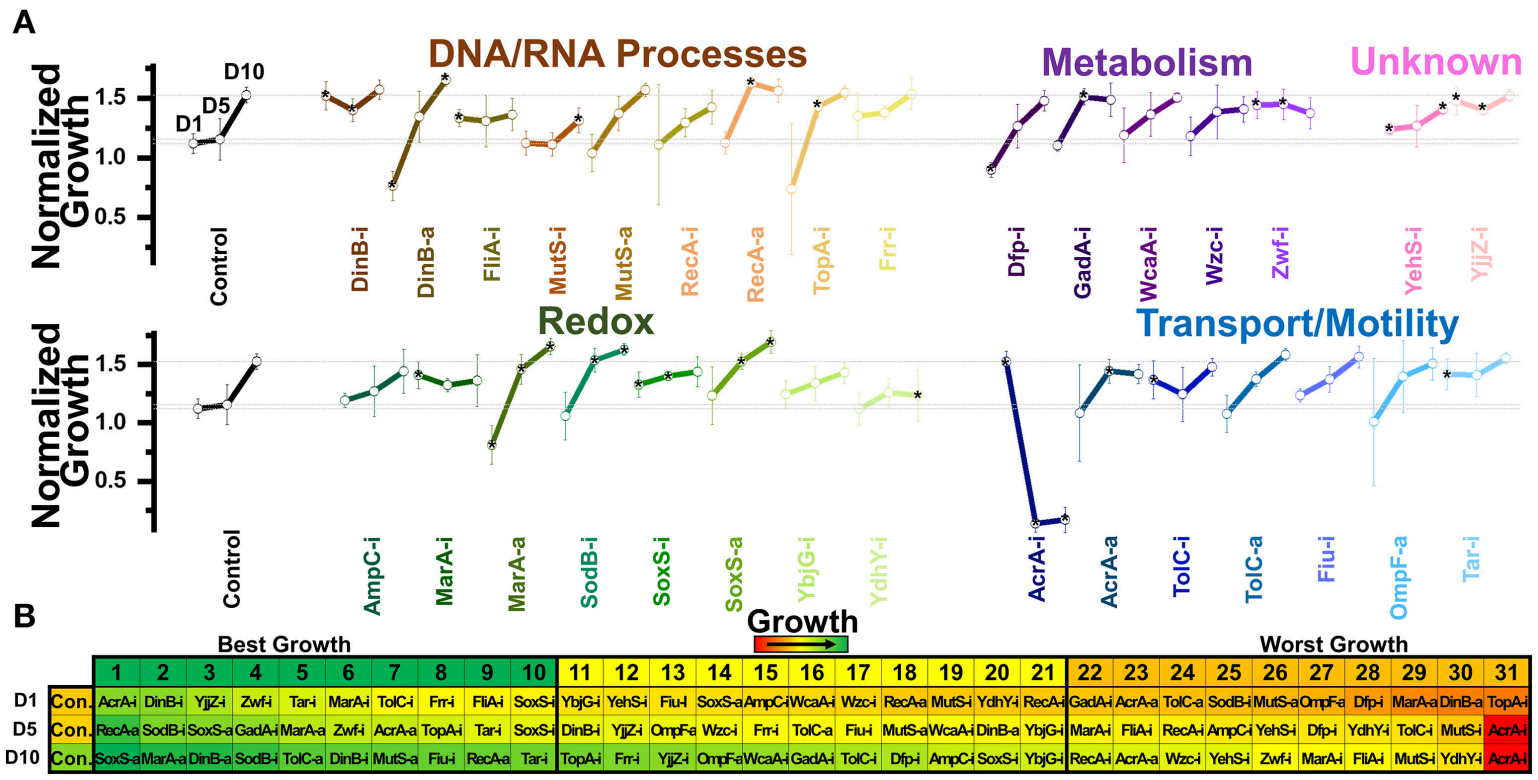
Normalized growth (maximum OD/starting OD) of *E. coli* harboring CRISPR gene perturbations during 10.0% vol/vol n-hexane exposure. **(A)** Change in the growth of each strain over 10 days of exposure, with quantification on days one (D1), five (D5), and ten (D10). Strains are organized based on pathways affected by perturbation. Dashed lines extend from the control for each experimental day. A two-tailed type II *t*-test was used to calculate significance (as indicated by *P < 0.05) relative to the control on the same experimental day. Error bars represent the standard deviation of four biological replicates. **(B)** Organized rankings of strains with highest growth reached on each day, with the color scale to indicate relative growth. The top 10 and bottom 10 are indicated as “best growth” and “worst growth,” respectively.

**Figure 6:**
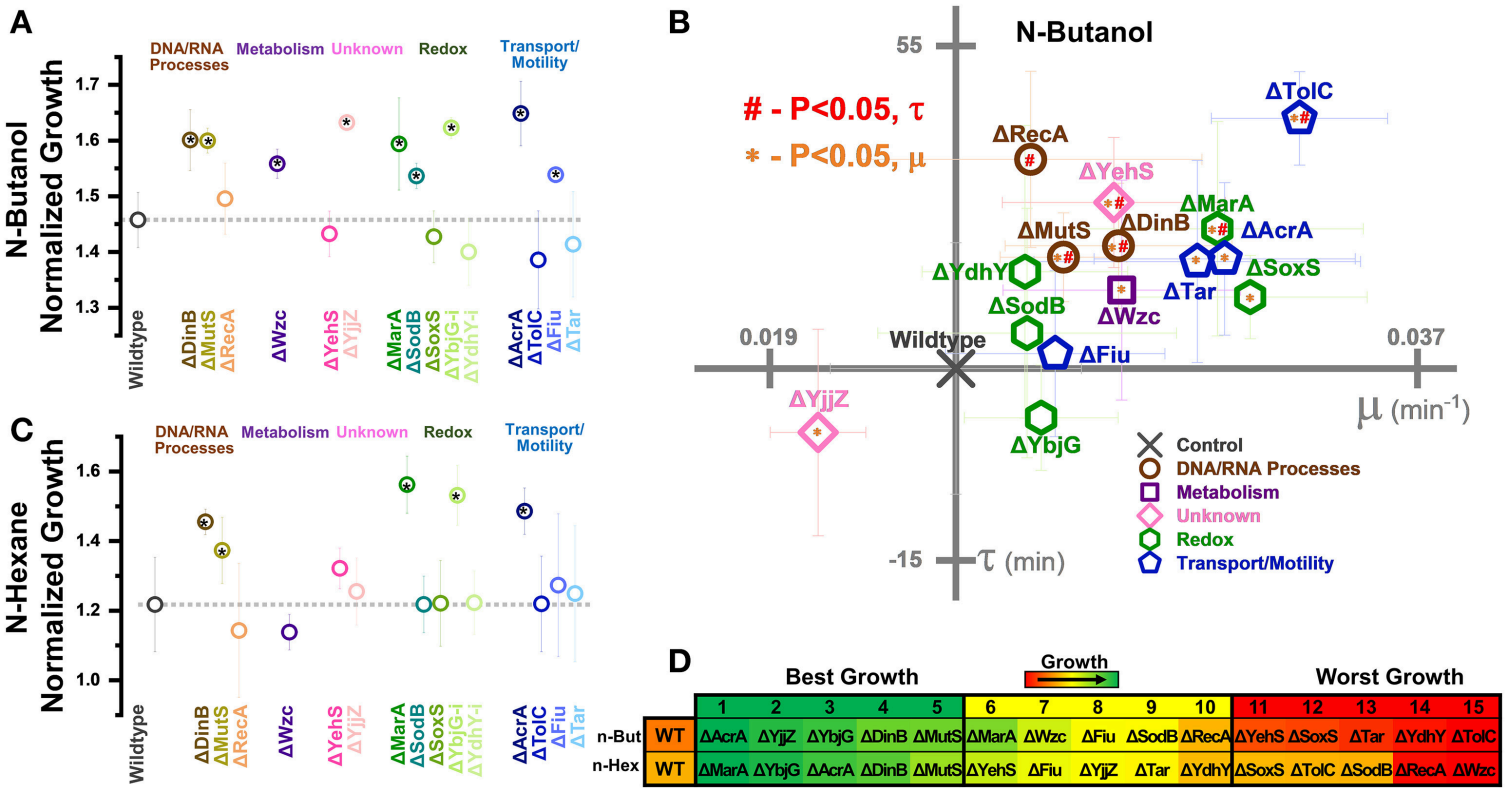
Growth of gene knockouts in relation to wildtype *E. coli* BW25113. Normalized growth (maximum OD/starting OD) of knockouts in **(A)** 0.5% vol/vol n-butanol or **(C)** n-hexane. Strains are organized based on pathways affected by perturbation. Dashed lines extend from the control **(A)** two-tailed type II *t*-test was used to calculate significance (*P* < 0.05) relative to the control. **(B)** Growth rates (μ) and lag times (τ) of knockouts during 0.5% vol/vol n-butanol exposure. Axes are set to intersect the control in each graph. A two-tailed type II *t*-test was used to calculate significance (*P* < 0.05) relative to the control in growth rates (orange *) and lag times (red #). **(D)** Organized rankings of strains with highest growth reached on each day, with the color scale to indicate relative growth. The top five and bottom five are indicated as “best growth” and “worst growth,” respectively. All error bars represent the standard deviation of four biological replicates.

For all growth experiments, individual colonies of normal CRISPR-perturbation constructs or gene knockouts were inoculated into 100 μL cultures in 384 well flat-bottom microplates and grown overnight for 16 h. Cultures were then diluted 1:100 into fresh 100 μL cultures supplemented with aTc (except for gene knockout strains) and grown for 24 h. Cultures were then diluted 1:100 into fresh 100 μL cultures supplemented with either no biofuel (Figure 2), 0.5% vol/vol n-butanol (Macron, Figures 3, 4, 6) or 10% vol/vol n-hexane (Macron, Figure 5), and grown in a GENios plate reader (Tecan Group Ltd.) operating under Magellan software (version 7.2) with shaking every 16.6 min before OD_580nm_ measurement every 20 min. Temperature was maintained at 37°C for this entire period. Cultures were grown for 24 h, and data from the microplate run was used to determine growth characteristics on “day 1” of the experiment. The significant volatility of n-hexanes disrupted optical density (OD) measurements during the first ~5 h of growth due to excessive evaporation onto the top of microplate lids causing significant condensation, hence the exclusion of lag times and growth rates for n-hexane data. For CRISPR perturbation strains, after 24 h of growth, cultures were diluted 1:100 into fresh media and grown in a regular shaking incubator for days two-four and six-nine. Cultures were again grown in the plate reader on days 5 and 10 of the experiment to capture changing growth characteristics over time.

**Figure 7:**
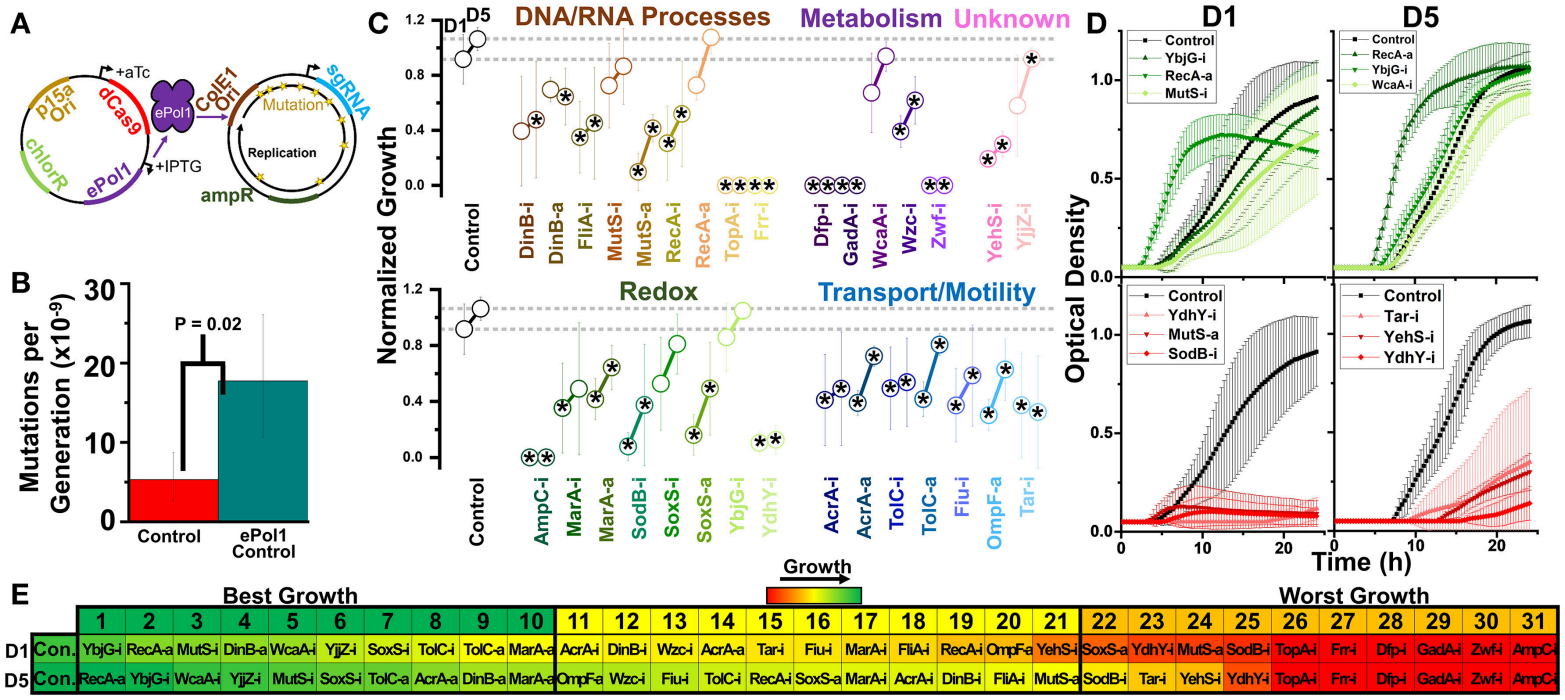
Design of a hyper-mutator strain of *E. coli* for targeted error-prone replication of the sgRNA plasmid, and subsequent growth of these strains in 1.0% vol/vol n-butanol exposure. **(A)** We move dCas9 and dCas9-ω onto a plasmid expressing IPTG inducible error-prone Pol1 in a strain of *E. coli* expressing temperature-sensitive native Pol1. During growth at 37°C, error-prone Pol1 is expressed, causing low fidelity replication of plasmids with the ColE1 ori. This imparts significant mutations of the sgRNA plasmids with minimal impact on the dCas9/dCas9-ω plasmid or genome at large. **(B)** Whole-genome mutation rates of the control strain and the hyper-mutator control strain. Error bars represent the standard deviation of 32 technical replicates. A two-tailed type II *t*-test was used to calculate the statistical difference between the strains. **(C)** Normalized growth (maximum OD/starting OD) of hyper-mutator *E. coli* harboring CRISPR gene perturbations during 1.0% vol/vol n-butanol exposure. Change in the growth of each strain over 5days of exposure, with quantification on days one (D1) and five (D5). Strains are organized based on pathways affected by perturbation. Dashed lines extend from the control for each experimental day. A two-tailed type II *t*-test was used to calculate significance (as indicated by *P < 0.05) relative to the control on the same experimental day. Error bars represent the standard deviation of four biological replicates. **(D)** Growth curves of the three strains growing to the highest levels (green, top) and lowest levels (red, bottom) on D1 and D5. **(E)** Organized rankings of strains with the highest growth reached on each day, with the color scale to indicate relative growth. The top 10 and bottom 10 are indicated as “best growth” and “worst growth,” respectively.

For Figure 7, four individual colonies of CRISPR-perturbation constructs expressing error-prone Pol1 in JS200 were inoculated directly from transformation plates into 100 μL cultures supplemented with amp and cm and grown for 16 h overnight. Cultures were diluted 1:100 into fresh 100 μL cultures supplemented with aTc, IPTG, and 1.0% vol/vol n-butanol (increased to exacerbate selective pressure) and grown for 24 h in the plate reader for day 1 growth measurements. OD measurements were repeated in the microplate reader for day 5 of the experiment. The increase in n-butanol concentration was done to drive further selection against CRISPR plasmids, while the reduced time of the experiment was due to our previous results showing similar growth phenotypes for most strains between days 5 and 10 of the experiment.

### Mutation fluctuation assay

Whole-genome mutation rates were determined using the Luria-Delbruck method of identifying spontaneous rifampicin resistance (Luria and Delbruck, 1943). An individual colony of strains for this experiment was inoculated in 3 mL of LB and grown overnight for 16 h without amp or cm selection. Each culture was then normalized to the same OD_595nm_ and grown, and diluted 1:10,000 into 35 parallel 100 μL cultures supplemented with 50 ng/mL aTc and grown for another 24 h. Three cultures of each strain were used to determine colony forming units, revealing overall viable cells per strain. Of the remaining 32 cultures, 50 μL of each were plated on LB agar supplemented with 100 μg/mL rifampicin (Sigma-Aldrich) and grown for 24 h. Colonies were then calculated, and mutation rates were estimated using the FALCOR web tool (Hall et al., 2009).

### Determination of sgRNA mutation rate via sequencing

To quantify mutation rates of the sgRNA plasmids in the error-prone polymerase system, twelve JS200 *E. coli* cells harboring error-prone Pol1 alongside dCas9-ω and the *ompF* activation sgRNA were exposed to 1.0% n-butanol for 5 days using the protocol listed above. After 5 days of exposure, replicates were streaked on plain LB plates and grown overnight. Sixteen individual colonies were selected from one replicate showing the greatest amount of growth, grown overnight in 5 mL LB, and miniprepped to recover the sgRNA plasmids. These plasmids were submitted for standard Sanger sequencing (GENEWIZ) using the primer 5′-aaataggcgtatcacgaggc-3′. Sequencing results revealed ~900 nucleotides of reliable sequence per sample. Mutations were identified via BLAST alignment, revealing a total of four point mutations in all 16 samples. From this data, it was estimated that four mutations per 900 ^*^ 16 nucleotides or a mutation profile of 2.78 ^*^ 10^−4^ mutations per nucleotide. As a 1:100 dilution of *E. coli* into fresh LB has been estimated to result in roughly ~6.64 new generations per day (Lenski et al., 1991), we estimate that 33.2 generations of bacteria passed throughout the 5-day evolution experiment. This gives an estimated mutation rate of 8.36 ^*^ 10^−6^ mutations per nucleotide per generation of the sgRNA plasmid. The established mutation rate of *E. coli* is 3.2 ^*^ 10^−9^ mutations per nucleotide per generation (Luria and Delbruck, 1943) (within error of our calculated mutation rate of the control in Figure 6B), suggesting that our system exhibited a ~2,600-fold increase in mutation rate. While this level is clearly higher than basal levels, it is significantly lower than the reported ~80,000-fold increase (Camps et al., 2003). This is likely due to a reduction in mutagenesis efficiency after reaching stationary phase, as has been reported (Alexander et al., 2014). Improved mutation rates could likely be achieved by maintaining cultures in exponential phase through growth in a bioreactor.

### Growth analysis

OD_580nm_ measurements were normalized to blank-LB cultures from the same day of each experiment. The resulting starting OD of each cutlure was then subtracted from each subsequent measurrement, thus normalizing growth to the starting timepoint. This was done in order to ensure accurate quantification of growth rates and lag times, as starting OD values were found to interfere with the program used to calculate these values. These final values were used to determine lag times, growth rates, and maximum ODs using the program *GrowthRates* version 1.8 (Hall et al., 2014).

For Supplementary Figures S1–S4, the normalized growth of all CRISPR perturbation strains in the absence of stress from Figure 2 were in turn normalized to growth of the control strain in the absence of stress. The change in growth that each CRISPR perturbation demonstrated relative to the control strain the absence of stress was calculated in Supplementary Figure S1. These relative growth values were used to normalize results from Figures 3A, 5A, 7C and Supplementary Figures S2–S4 respectively by multiplying these results to the output shown in Supplementary Figure S1.

### Batch culture growth

The four replicates of the five strains most tolerant to n-butanol, as well as the control strain perturbing *rfp*, were saved as glycerol stocks at the end of 10 days of 0.5% n-butanol exposure. Stabs of these glycerol stocks were used to inoculate 3 mL LB cultures supplemented with amp, cm, aTc, and 0.5% n-butanol, and grown overnight for 16 h in a 37°C incubator with continuous shaking at 225 rpm. Overnight cultures were then diluted 1:100 into fresh 15 mL cultures supplemented with amp, cm, aTc, and 0.5% n-butanol. A 200 μL aliquot of each culture was then collected to determine starting ODs in a microplate, while the remaining culture was grown in a 37°C incubator with continuous shaking at 225 rpm. After 6 h of growth (i.e., mid-log phase), another 200 μL aliquot of each culture was collected to determine ODs in a microplate. The rest of the culture was grown for another 18 h and used for RT-qPCR.

### Quantitative reverse transcription PCR

The majority of constructs' impact on bacterial gene expression were confirmed in previous studies to perturb mRNA levels in a 10-fold range above or below basal levels (Erickson et al., 2015, 2017; Otoupal et al., 2017). To confirm that perturbations remained effective over prolonged periods, RT-qPCR was performed on the samples collected in the Batch Culture Growth section above. Three of the four replicates of each strain was collected at the end of 24 h of growth, and RNA was extracted using the GeneJET RNA Purification Kit (Thermo Scientific). The collected RNA was diluted to a concentration of 5,000 ng per 100 μL, and was purified for any DNA contamination with the TURBO DNA-free kit (Ambion). Purified 100 ng of RNA was subsequently converted into 100 ng of cDNA through the Maxima First Strand cDNA Synthesis Kit for RT-qPCR (Thermo Scientific). RT-qPCR reactions were then performed on technical duplicates of each replicate using the Maxima SYBR Green qPCR Master Mix (Thermo Scientific). Two ng of cDNA were used in 25 μL RT-qPCR reactions, which were run on the QuantStudio 6 Flex Real-Time PCR System (Applied Biosystems). Forty cycles of 98°C melting for 15 s, 50°C annealing for 30 s, and 72°C extension for 30 s. Rox normalization was applied across the plate, after which Ct values were estimated and averaged across technical duplicates. Gene expression changes were calculated using the 2^−ΔΔCt^ approach with the genes *gyrA* and *cysG* serving as separate housekeeping gene controls. Application of the 2^−ΔΔCt^ approach was done for both housekeeping genes separately, relative to the ΔCt expression of the control strain perturbing *rfp*. Averages of fold changes in gene expression were taken from both housekeeping genes. Controls were also included, using either no cDNA in the reaction or using an RNA sample for which cDNA was prepared with no reverse transcriptase present.

### Statistical analysis

All *P* values reported were calculated using a standard two-tailed type II student's *t*-test in comparison to the RFP-targeting control strain within each graph, with a significance value of α = 0.05. All normalized growth, optical density, growth rate, and lag time error bars represent standard deviations of four or eight biological replicates as indicated. Error bars of mutation fluctuation analysis represent standard deviations of 32 technical replicates.

## Results

### Construction of CRISPR perturbations and quantification of impact on *E. coli* growth during no biofuel exposure

We first designed a diverse library of 31 CRISPR perturbations to modulate gene expression in *E. coli*. These gene targets were selected based on previous genes known to be involved in general bacterial stress response (*acrA, dinB, marA, mutS, recA, soxS*, and *tolC*) (Otoupal et al., 2017), or to be involved in central biological processes (*dfp, frr, gadA, topA*, and *zwf*) (Sardessai and Bhosle, 2002; Dunlop, 2011; Erickson et al., 2014). We also explored genes that we had previously identified to exhibit altered transcriptomic signatures during exposure to n-butanol or n-hexane (Erickson et al., 2017) (*tar, fliA, fiu, wcaA, wzc, ybjG, ydhY, yehS*, and *ybjG*). *OmpF* has been associated with improved solvent tolerance (Isken and de Bont, 1998), and *ampC* expression has been directly correlated to diminished *ompF* activity (Pérez et al., 2007). Finally, *sodB* overexpression has demonstrated significantly increased n-butanol tolerance (Reyes et al., 2013). These genes were selected over other genes associated with biofuel tolerance as they are not co-transcribed with other genes, or are contained in relatively small operons, so as to minimize the CRISPR perturbation's direct impact on other genes.

CRISPR inhibition constructs were designed to repress gene expression by binding within the first ~50 nt of the open reading frame, or around the +1 site of the respective promoter (Larson et al., 2013). CRISPR activation constructs were designed to bind ~80–100 nt upstream of the +1 site of the promoter. These gene perturbations resulted in decreasing or increasing mRNA production to ~10-fold basal levels as shown in previous studies (Erickson et al., 2015, 2017; Otoupal et al., 2017).

We first tested how these strains behaved during growth in plain LB, to demonstrate how perturbations impacted growth in the absence of stress (Figure 2). We quantified the maximum growth each strain reached at the end of 1 day of growth (Figure 2A) and plotted the growth curves of the fastest and slowest growing three strains as ranked by maximum growth reached (Figure 2B). We also determined the growth rates and lag times of each strain in relation to the control (Figure 2C).

Out of 31 perturbation strains, only six showed significant differences in growth. Only strain DinB-a grew significantly better than the control. Five strains (RecA-i, TopA-i, WcaA-i, AmpC-i, and OmpF-a) grew to significantly lower concentrations than the control. Only TopA-i grew slower than the control. Two strains (MarA-a and SodB-i) exhibited a longer lag time, while five strains (RecA-a, TolC-a, AcrA-a, DinB-a, and Wzc-i) exhibited shorter lag times. All five of these strains exhibiting shorter lag times were also in the top ten strains in terms of ranked growth (Figure 2D), suggesting a slight inherent benefit to gene activation on *E. coli* growth.

Overall, these data demonstrate that growth of perturbed strains in plain LB medium was at most moderately disrupted by gene perturbations. This is most noticeable by observing the overall growth curves of the top and bottom-growing strains, which reveal similar growth trajectories relative to the control (Figure 2B). Any noted difference from the control of each perturbed strain was taken into consideration in future analysis. A summary of every strains' growth in the presence of biofuel stress relative to its growth in the absence of stress is presented in Supplementary Figures S1–S4.

### Impact of CRISPR perturbations on *E. coli* growth during n-butanol exposure

We next exposed our CRISPR perturbations to 0.5% vol/vol n-butanol and analyzed how each perturbation impacted growth. This experiment was performed over the course of 10 days, with 1:100 dilution into fresh media at the start of each day. Growth was quantified in a microplate reader on days 1, 5, and 10 of the experiment. We again analyzed the normalized growth of each strain on each day of the experiment (Figure 3A) and plotted the growth curves of the top three and bottom three strains on each day of the experiment (Figure 3B). We also ranked each perturbation by maximum growth reached during each day, parsing out the top 10 strains exhibiting the highest and lowest growth respectively (Figure 3C). Perturbations were compared against the nonsense targeting RFP control strain.

The most prominent growth impacts were observed from metabolic pathway perturbations. Strains Dfp-i, Zwf-i, and GadA-i always fell within the 10 worst performing strains. Each of these genes is involved in central metabolic pathways—*dfp* is essential for coenzyme A synthesis, *zwf* expresses the first enzyme of the pentose phosphate pathway, and *gadA* regulates glutamate levels. Disruption of such central metabolic pathways appears to be deleterious to butanol tolerance across all time points. Conversely, inhibition of *wzc* exhibited the highest growth on day 1 and was always one of the top three strains throughout the experiment. Growth curves for this strain demonstrated significant improvement over the control on all days of the experiment (Figure 3B). Interestingly, we observed that Wzc-i also demonstrated a ~9-fold increase in chromosomal mutation rates (Supplementary Figure S5).

Intriguing time-dependent impacts on growth were observed from perturbations of transport and motility genes. Strain AcrA-a exhibited detrimental growth on day one, but improved growth later in the experiment, while strain AcrA-i. exhibited reduced growth only on day 10. TolC-a and Fiu-i showed improved growth at the beginning of the experiment but had no significant impact by day 10. Tar-i helped growth on day 1 but actually resulted in lower growth later in the experiment. Activation of *ompF* also resulted in diminished growth in the beginning, but this could be explained by the aforementioned diminished growth in the absence of n-butanol.

Very strong growth impacts were observed by a few perturbations of DNA and RNA processes. This is best demonstrated by activation of *dinB* and *recA*, which both exhibited improved growth, while inhibition of these genes decreased growth. Perturbations of *mutS* exhibited the opposite effect, with inhibition improving growth and activation decreasing growth. TopA-i and Frr-i exhibited time-dependent phenotype switching as many of the transport gene perturbations did, both slightly improving growth in the beginning while resulting in diminished growth at later time points. The complex phenotypic responses of these perturbation strains highlight the transitory impacts of CRISPR perturbations on overall growth.

Relative to other perturbations, those impacting redox pathways exhibited less of an impact on growth phenotypes. The exception to this is activation of *marA*, which grew to the fourth and second highest OD on the days 5 and ten. Interestingly inhibition of *marA* also improved growth on day 1. *MarA* has been demonstrated to be significantly upregulated during n-butanol exposure (Rutherford et al., 2010), so while results pertaining to MarA-a are as expected, MarA-i improved growth are intriguing.

Finally, perturbation of the uncharacterized genes *yehS* and *yjjZ* point to the untapped potential for improved n-butanol tolerance and need for further investigation. Our previous work analyzing the transcriptome of *E. coli* adapted to n-butanol revealed that *yjjZ*, an uncharacterized gene suggested to express a small RNA, was significantly downregulated during exposure to n-butanol (Erickson et al., 2017). In accordance with this, strain YjjZ-i grew to the second highest levels on day one. However, this perturbation appears to have provided no benefit in the later part of the experiment. Conversely, strain YehS-i exhibited improved growth on days 5 and 10.

Overall, we noted that all strains adapted to n-butanol exposure over time; the average maximum ODs increased (0.65 ± 0.15 and 0.73 ± 0.19 on day 1 and 10, respectively). The impact of gene expression perturbations appears to be significantly time-sensitive, with many exhibiting benefits only in the short-term that were lost in the long-term. Supporting this observation is the fact that the control strain became one of the best strains over time; the control strain grew to the fifth lowest levels on day 1 but 13 highest on day 10.

In addition to testing bacterial tolerance to n-butanol in microplates, we also tested the tolerance of some of the best performing strains in larger batch cultures. This was done to demonstrate the feasibility of translating these results to larger volumes that would be required when applying these gene expression perturbations in an industrial setting. We found that of the five best performing strains in n-butanol on day 10 of the experiment, all but strain MutS-i maintained statistically significant improved growth over the control strain in 15 mL batch cultures (Supplementary Figure S6). Additionally, RT-qPCR of these cultures revealed that each of them (with the possible exception of Wzc-i) maintained gene perturbations after 10 days of n-butanol exposure, as well as after the re-exposure (Supplementary Figure S7).

### Impact of CRISPR perturbations on *E. coli* lag times and growth rates during n-butanol exposure

We also characterized the perturbations' impacts on lag times and growth rates on day 1 (Figure 4A), day 5 (Figure 4B), and day 10 (Figure 4C). These results could point to interesting differences in growth between the perturbation strains upon exposure to n-butanol stress.

Interestingly, while growth was generally improved by perturbations relative to the control on day 1, the opposite was true for lag times, which were generally extended: Twenty six strains exhibited significant increases in lag times (Figure 4A). Across the entire experiment, only four strains (Zwf-i, TopA-i, Tar-i, and Frr-i) consistently demonstrated increased lag times (Figures 4B,C). The best performing strain, DinB-a, began to exhibit decreased lag times at later time points.

Many of the strains growing to the lowest levels also grew the slowest. Six of the 10 worst growing strains on day 1 (Dfp-i, OmpF-a, AcrA-a, FliA-i, AmpC-i, and Zwf-i), six of the 10 worst growing strains on day 5 (GadA-i, Frr-i, Dfp-i, MarA-i, DinB-i, and RecA-i), and the worst growing strain (RecA-i) on day 10 all grew significantly slower on their respective days. Conversely, many of the best growing strains also grew the fastest. This includes six of the best ten growing strains on day 1 (YjjZ-i, Frr-i, TolC-a, Wzc-i, RecA-a, and DinB-a), 4 on day 5 (YehS-i, DinB-a, YbjG-i, and Wzc-i), and 6 on day 10 (DinB-a, YbjG-i, RecA-a, YehS-i, Wzc-i, and AcrA-a).

Of note is the impact of gene expression activations over time. While lag times were relatively similar between both inhibition and activation constructs, gene activations appeared to grow particularly faster over time. On day one, three activations improved growth rates while another three decreased growth rates. No gene activation slowed growth on days 5 and ten, while all gene activations aside from MarA-a and TolC-a significantly increased growth rates on day 10.

### Impact of CRISPR perturbations on *E. coli* growth during n-hexane exposure

One of the strong benefits of CRISPR gene perturbations is that it is relatively easy to test under diverse conditions, as everything is expressed from stable plasmids. We therefore performed the same growth assays of our CRISPR strains during exposure to a different biofuel, n-hexane, to demonstrate the power of this approach to identify gene targets under diverse conditions. We again quantified growth during the first, fifth, and tenth day of exposure to 10% vol/vol n-hexane (Figure 5). Due to the high-volatility of n-hexane, OD measurements were obscured during the initial few hours of growth, rendering lag time and growth rate calculations unreliable. Maximum ODs were still able to be measured in later hours of the experiment, allowing for determination of normalized growths (Figure 5A).

Perturbations related to nucleic acid processes resulted in diverse responses across time points. This is most aptly demonstrated by strains DinB-i and DinB-a, which were the 2nd best and 2nd worst growers on day 1, respectively. DinB-i growth stayed virtually constant over the experiment. Conversely, DinB-a growth steadily improved, and eventually exhibited the third highest growth. Strains FliA-i, RecA-a, and TopA-i also showed transitory improvements in growth that only emerged on days 1 or five. The impact of *mutS* inhibition also appeared to be time-sensitive, having little impact on the first day but growing to the 2nd and 3rd lowest levels on day 5 and 10, respectively. Collectively, controlling the expression of nucleic acid processes appears to be highly time sensitive.

Metabolic perturbations resulted in a less pronounced impact on growth in n-hexane than they showed in n-butanol. Only inhibition of *dfp* on day 1 exhibited diminished growth but was able to recover on latter days. Inhibition of *gadA* and *zwf* appeared to provide a short-term benefit, but this too was lost by the final day of the experiment. This suggests that manipulation of metabolic pathways has less potential for optimization of n-hexane tolerance.

Conversely, perturbation of redox pathways elicited greater growth changes in n-hexane than was observed in n-butanol. Inhibition of both *marA* and *soxS* improved growth on day one, while activation of *marA* resulted in the third-lowest growth. This trend was notably reversed on days 5 and ten, with activation of these genes significantly improving growth. Indeed, these two strains were the best growing strains by the final day of the experiment, with the inhibited strains improving very little over time.

Another interesting result is the inhibition of *sodB*, which grew to the 2nd and 4th highest levels on day 5 and 10, respectively. A potential explanation for this phenomenon could be the degradation of n-hexane into n-hexanol in *E. coli* related to oxide levels, catalyzed by *sodB*'s gene product - superoxide dismutase. Alcohols are typically more toxic than alkanes due to their higher polarity (Sardessai and Bhosle, 2002), and reduced *sodB* expression could disrupt conversion into this more toxic chemical.

Most perturbations of transport-related genes had little impact on growth, with the prominent exception of *acrA* inhibition. This strain exhibited the highest optical densities after the first day of n-hexane exposure, but every replicate died by the fifth day of the experiment (Figure 5B). The AcrAB-TolC efflux pump is known to export solvents such as hexanes from inside *E. coli* (Takatsuka et al., 2010). The eventual death caused by *acrA* inhibition demonstrates that engineered transcriptome changes are sufficient to mimic total gene knockout phenotypes. The established connection between this efflux pump and n-hexane tolerance explains the higher ODs upon activation of *acrA* on day 5, but runs counter to *tolC* perturbation results wherein activation never significantly impacted ODs, and whose inhibition also increased ODs on day 1.

Finally, inhibition of the uncharacterized genes *yehS* and *yjjZ* during exposure to n-hexane resulted in similar phenotypes as observed in n-butanol—both perturbations improved growth on day one, with *yjjZ* demonstrating the third highest growth. As perturbation of these genes was again able to improve biofuel tolerance, our data indicate that these genes are highly promising candidates for future research.

### Gene knockout phenotypes corroborate CRISPR perturbation results

To corroborate our CRISPR perturbation results, we examined the growth of fifteen gene knockouts in the presence of 0.5% n-butanol (Figure 6A) and 10.0% n-hexane (Figure 6C) exposure. This included three genes related to DNA/RNA processes (*dinB, mutS*, and *recA*), the metabolism gene *wzc*, the unknown genes *yehS* and *yjjZ*, five redox-related genes (*marA, sodB, soxS, ybjG*, and *ydhY*), and four transport or motility-related genes (*acrA, tolC, fiu*, and *tar*).

We first examined the growth of these knockout strains in the absence of biofuel stress (Supplementary Figure S8). Removal of both uncharacterized genes *yehS* and *yjjZ* resulted in significant increase in normalized growth, as did removal of *ydhY*. A slight reduction in growth was observed by *soxS* removal. None of these four knockouts resulted in significant shifts in growth in a CRISPR knockdown context. Also of note was a reduction in growth rates and increase in lag time caused by knockout of *recA*.

During exposure to biofuels, nine knockouts exhibited significant increases in growth over the wildtype during n-butanol exposure. Of these, seven (Δ*mutS*, Δ*wzc*, Δ*yjjZ*, Δ*sodB*, Δ*ybjG*, Δ*acrA*, and Δ*fiu*) exhibited similar phenotypes as the corresponding CRISPR perturbations. While Δ*dinB* improved growth, activation of *dinB* gene expression also resulted in improved growth. In a similar vein, Δ*marA* resulted in improved growth.

Interestingly, Δ*yjjZ* actually exhibited the slowest growth rate in n-butanol (Figure 6B). This runs contrary to the CRISPR perturbation results, where its inhibition resulted in the fastest growth rate of all strains on day 1 (Figure 4A), and was not observed in the absence of biofuel stress. Of the remaining gene knockouts, nine exhibited significant increases in growth rates relative to the control, five of which (Δ*acrA*, Δ*marA*, Δ*wzc*, Δ*dinB*, and Δ*mutS*) also exhibited increased growth over the control. Only three of these strains (MutS-i, Tar-i, and YehS-i) actually exhibited an increased growth rate in the CRISPR perturbation context. The remaining four strains (Δ*yehS*, Δ*tolC*, Δ*soxS*, and Δ*tar*) were four of the five worst growing strains (Figure 6D).

Six strains exhibited increased lag times in n-butanol (Δ*recA*, Δ*mutS*, Δ*yehS*, Δ*dinB*, Δ*marA*, and Δ*tolC*), and no strain exhibited decreased lag time. While CRISPR inhibitions of each of these strains also exhibited increased lag times on day 1, it should be noted that most CRISPR perturbation strains increased lag times over the control. This trend was broadly recapitulated in gene knockouts, suggesting that an underlying phenomenon is indeed causing these genetic manipulations to increase lag times during n-butanol exposure.

Growth in n-hexane was improved by five knockouts (Δ*dinB*, Δ*mutS*, Δ*marA*, Δ*ybjG*, and Δ*acrA*), of which all but MutS-i and YbjG-i improved growth in the CRISPR perturbation context (Figure 6C). None of these knockouts improved growth in the absence of biofuel stress. The strongest improvements in n-hexane tolerance were again related to redox-related genes, as Δ*marA* and Δ*ybjG* were the top two growing strains (Figure 6D). The large improvements in growth observed from SodB-i only emerged in later time points and could be why Δ*sodB* showed no significant differences from the control.

### CRISPR perturbations retain growth impacts despite a hyper-mutator phenotype

One frequent criticism of CRISPR perturbation strategies is the potential for mutations to arise that inactivate the system. As bacteria are continually exposed to stressful conditions, they inevitably accumulate mutations; a mutation in the CRISPR expression system, such as a deletion in the sgRNA, could deactivate the perturbation. This is especially concerning if the perturbation is detrimental at any point during growth, which we have demonstrated is frequently the case. To address these concerns, we designed a system that biases the sgRNA plasmid toward hyper-mutation rates to illustrate how mutation rates can affect the efficacy of CRISPR perturbation strategies.

We accomplished this by incorporating an error-prone version of Polymerase 1 (Pol1) with greatly diminished fidelity into our CRISPR perturbation strains on the plasmid expressing dCas9 (Figure 7A, see Methods). We transformed each CRISPR construct into a strain of *E. coli* with temperature-sensitive wild-type Pol1 that fails to express at temperatures above 30°C, causing this error-prone version of Pol1 to overtake its functionality. Pol1 initiates replication of ColE1 plasmids, while having no role in replicating plasmids using the pSC101 ori that drives dCas9 and dCas9-ω expression. Thus, the sgRNA plasmid is significantly more prone to accumulating mutations in this system. Previous work designing this error-prone Pol1 estimated that *in vivo* mutation rates are increased ~80,000-fold above basal levels for at least 3 kb beyond the ColE1 ori (Camps et al., 2003), with only 3 to 5-fold increases in mutation rates of the chromosome at large. We confirmed that integration of the error-prone Pol1 into our bacteria increased chromosomal mutation rates ~3-fold (Figure 7B), in line with these published results.

By incorporating error-prone Pol1 into our CRISPR perturbation system, we could simulate how prolonged mutation might impact the efficacy of our perturbations toward engineering biofuel tolerance. We implemented this system into each of our CRISPR perturbation strains, and again tested their impacts on growth during biofuel exposure. We focused on n-butanol stress due to the aforementioned difficulty of quantifying growth rates and lag times in n-hexane. n-Butanol was doubled to 1% vol/vol to increase the selective pressure driving mutations. As the majority of impacts in n-butanol emerged by day 5 of the experiment, we limited the experiment to 5 days of exposure, quantifying growth phenotypes on the first and last day (Figure 7C and Supplementary Figure S9). Finally, we included ampicillin selection to ensure that the sgRNA plasmids were not lost completely due to mutations, thereby biasing mutations solely toward the portion of the plasmid responsible for expressing the sgRNA.

Of immediate note is the failure of six strains to grow at this higher concentration of n-butanol. That these strains were unable to recover by day 5 suggests that even the hyper-mutation rates of this system were not sufficient to recover the detrimental phenotype, and suggest short-term stability of the CRISPR perturbation system even when causing reduced fitness. These strains harbored inhibition constructs targeting the genes *ampC, gadA, dfp, zwf, topA*, and *frr*. Each strain was one of the 10 worst growing strains on day 1 (Dfp-i, AmpC-i, GadA-i, and Zwf-i), day 5 (Dfp-i, Zwf-i, TopA-i, Frr-i, and GadA-i), or day 10 (Dfp-i, GadA-i, and Zwf-i) at lower n-butanol concentrations in the absence of the hyper-mutator phenotype, suggesting that this is a result of doubling butanol levels. Half of these genes were metabolism-related (*dfp, gadA*, and *zwf*). Two of these other genes, *topA* and *frr*, are essential for growth, and their inhibition likely synergized with the toxic effects of butanol to induce cell death. The final gene, *ampC*, is an inherent periplasmic beta-lactamase, and to our knowledge has never been linked to biofuel tolerance. The death of these six strains suggests that expression of these genes should strongly be considered when optimizing n-butanol tolerance.

The control strain was the top performing strain on day 1 and second best on day 2, indicating perturbations were largely detrimental at this increased n-butanol concentration. We plotted the growth curves of the top and bottom three perturbation strains on each day, excluding the six strains that died during growth (Figure 7D). RecA-a growth curves strikingly show a significantly faster growth rate and reduced lag time (confirmed in Supplementary Figure S9), despite not reaching higher concentrations at the end of each day.

Overall, most detrimental perturbations did not reach control level growth after 5 days of exposure (Figure 7E). This suggests that despite the hypermutator phenotype, detrimental phenotypes remained detrimental. To ensure that this was not a result of the failure of the hyper-mutator phenotype, we sequenced 16 individual colonies of the moderately detrimental OmpF-a perturbation. We observed four mutations in the sgRNA plasmid, none of which were located in the actual sgRNA coding sequence. This led us to estimate a mutation rate of 8.36 ^*^ 10^−6^ mutations per nucleotide per generation, or a ~2,600-fold increase in mutation rates above basal levels (see Methods). While this estimate is significantly lower than the 80,000-fold increase reported for the error-prone Pol1 system, we can confidently report a large increase in mutation rates. Collectively, this data demonstrates that CRISPR perturbations are stable even in a hyper-mutator strain.

## Discussion

This study applies recent advances in synthetic biology to harness the untapped potential of altering gene expression states in biofuel applications. We explored 31 unique CRISPR inhibitions and activations of a diverse set of bacterial genes and quantified their impacts on *E. coli* growth during exposure to two common biofuels, n-butanol and n-hexane. We identified a number of strong gene candidates whose expression could be engineered to enhance biofuel tolerance such as RecA-a, YjjZ-i, and Wzc-i.

A number of these perturbations' growth impacts were time-sensitive, suggesting that they could be implemented into temporal gene circuits to improve biofuel production capacity. This possibility is gaining popularity due to the relatively facile ability to integrate CRISPR perturbations into such circuits (Cress et al., 2016; Wiktor et al., 2016). Indeed, as efforts to improve biofuel tolerance have stalled, the need for genetic circuits to manipulate transcription at particular time points has been recognized yet relatively unexplored (Dunlop et al., 2010). The complex impacts of perturbations of transport and motility genes we observed during n-butanol exposure could explain why previous attempts to improve *E. coli* n-butanol tolerance by heterologously expressed efflux pumps have not been successful (Dunlop et al., 2011), as the fitness impact of these genes appears to depend on time. Furthermore, the extended lag times we observed from CRISPR activated strains during n-butanol exposure suggest that greater tolerance can be made by waiting to activate gene expression until after bacteria have adjusted to butanol exposure. Finally, the apparent improvement of growth caused by *acrA* and *tolC* inhibition during the early stages of n-hexane exposure suggests that temporal manipulation of the AcrAB-TolC efflux pump could offer an interesting strategy to improve bacterial tolerance to alkanes. The need to optimize expression in a time-sensitive manner is becoming more apparent, and CRISPR perturbation can make such genetic circuits attainable. This study presents the first evidence toward this goal.

This study also presents evidence that particular pathways are more appealing for CRISPR perturbation to optimize bacterial tolerance to biofuels. For instance, manipulation of central metabolic pathways produced particularly pronounced effects on n-butanol tolerance, such as improved growth during *wzc* inhibition. *Wzc* is involved in colanic acid biosynthesis (Stevenson et al., 1996), and these results suggest that diverting metabolic flux from colanic acid could improve growth in n-butanol. The heightened mutation rate of this strain could additionally explain its consistent improvement in n-butanol tolerance. In a similar vein, the responsiveness of *E. coli* to redox perturbations in n-hexane suggests a potential area of focus for improving n-hexane tolerance.

Engineering gene expression has long been a goal for biotechnology application. However, previous approaches for accomplishing this including manipulation of promoter elements (Bordoy et al., 2016) or riboswitches (Berens et al., 2015) have each suffered from their own unique drawbacks that have made them difficult to implement in practice. Perhaps the most notable limitation is the reliance upon stable alteration of genomes. CRISPR perturbations, on the other hand, can be implemented without direct manipulation of the bacterium's genome via plasmids or extracellular delivery of the CRISPR machinery. This can also be much easier to engineer in practice than direct mutations of the genome, which has frequently proven difficult in a number of promising biofuel producing microorganisms (Hsu et al., 2014). Here we have shown that simple knockdowns can be sufficient to impart significant growth phenotypes that mimic total gene removal.

Furthermore, multiplexing sgRNAs to target one gene multiple times or multiple genes at the same time is exceedingly simplified by the introduction of numerous unique sgRNAs simultaneously and is gaining significant attention (Zalatan et al., 2014; Cress et al., 2015a). Combining the best perturbations presented in this study could conceivably be done to raise tolerance levels even further. On the other hand, we observed a trend over time during n-butanol and n-hexane exposure in which the control strain appeared to become one of the more tolerant strains by day 10 of the experiment. This could suggest that CRISPR gene expression perturbations slightly impeded the strains' adaptive potential in the long-term. This is supported by previous work that has noted how epigenetic epistatic interactions might constrain adaptation (Park and Lehner, 2013; Chou et al., 2014; Otoupal et al., 2017, 2018), and could imply a tendency for perturbations to be detrimental to improving butanol tolerance in the long-term. This would again support the notion that genetic circuits that induce perturbations only after adaptation occurs is a promising path toward enhanced bacterial biofuel tolerance.

We also demonstrated that despite artificially amplifying sgRNA mutation rates ~2,600-fold, CRISPR perturbations induced similar growth phenotypes. We showed that no mutations arose inactivating the perturbation after 5 days, suggesting that spontaneous mutations inactivating the system are less likely than might be initially predicted. CRISPR perturbations appear to be able to be maintained stably for prolonged periods without loss of functionality, which is further supported by RT-qPCR results after 11 days of n-butanol exposure. As such perturbations begin to be applied toward biotechnology purposes, such long-term stability will be essential to maintain the desired phenotype. Our data suggest that loss of sgRNA functionality, even if detrimental, is unlikely in the short to medium term.

It should be noted that a few of the gene knockouts failed to replicate CRISPR perturbation results. This is particularly apparent in exploration of *dinB* and *marA*. While our *dinB* activation results are consistent with previous work showing that *dinB* overexpression improved long-term adaptive potential toward n-butanol (Zhu et al., 2015), a knockout of the gene also improved growth. Furthermore, both *marA* knockout and activation resulted in improved growth n-butanol. The rapid over-expression of *marA* immediately after n-butanol exposure has been previously reported (Rutherford et al., 2010). However, it has also been demonstrated that total knockout of *marA*'s repressor, *marR*, resulted in diminished growth in n-butanol (Luhe et al., 2012). A potential explanation for these conflicting methods of improved n-butanol tolerance could stem from the fact that the *marRAB* operon is known to exhibit stochastic pulsing behavior (Garcia-Bernardo and Dunlop, 2013). The fact that these knockouts demonstrated such counterintuitive results indicates that their influence on n-butanol tolerance may be more nuanced than a simple “on-off” response. These genes are promising candidates for further study for biofuel tolerance, particularly in a potential genetic circuit context.

Going forward, we envision that this hyper-mutation system could be employed toward the directed evolution of novel sgRNA targets, improving fitness without requiring *a priori* knowledge. Over long periods, detrimental mutations to the sgRNA would be selected against, while the rare beneficial mutations that redirect CRISPR perturbations to new targets would be selected for in a manner highly analogous to traditional directed evolution approaches (Alper and Stephanopoulos, 2007). Various alterations could be made to our hyper-mutator system to make this approach more viable. For instance, it has been reported that mutagenesis from error-prone Pol1 is strongest during stationary phase, and suggested that mutations are concentrated in locations closest to the origin of replication (~700 bp) (Camps et al., 2003; Alexander et al., 2014). Removal of extraneous DNA segments would increase the likelihood of targeted mutations toward the 20 nt target sequence of the sgRNA. It may be beneficial to express the sgRNA in its native, two component fashion where tracrRNA is expressed separately from the target sequence: expressing the tracrRNA on a separate plasmid would ensure its structure is not lost by mutation. Growth in a bioreactor to maintain steady-state conditions would ensure maximum mutation rate, and would likely impart a more consistent selective pressure to obtain beneficial mutations. This would also allow for a controlled increase of butanol concentration, as the constant butanol concentration used in this study likely limited further selection.

## Author contributions

PO designed the study, performed all experiments, and analyzed all data. PO and AC wrote and revised the manuscript. All authors read and approved the manuscript.

### Conflict of interest statement

The authors declare that the research was conducted in the absence of any commercial or financial relationships that could be construed as a potential conflict of interest.

## Abbreviations

CRISPR: clustered regularly interspaced short palindromic repeats
dCas9: deactivated CRISPR-associated protein 9
dCas9-ω: fusion of dCas9 with omega subunit of RNA polymerase
sgRNA: single guide RNA
CRISPRi: CRISPR interference (of gene expression)
CRISPRa: CRISPR activation (of gene expression)
ORF: open reading frame
RFP: red fluorescent protein.
