# Supplementary material for: CRISPR Gene Perturbations Provide Insights for Improving Bacterial Biofuel Tolerance
Source: Front Bioeng Biotechnol. 2018 Sep 4;6:122. doi: 10.3389/fbioe.2018.00122 (PMC6131188; doi:10.3389/fbioe.2018.00122)
Supplement: Supplementary file 1 [file Data_Sheet_1.pdf]

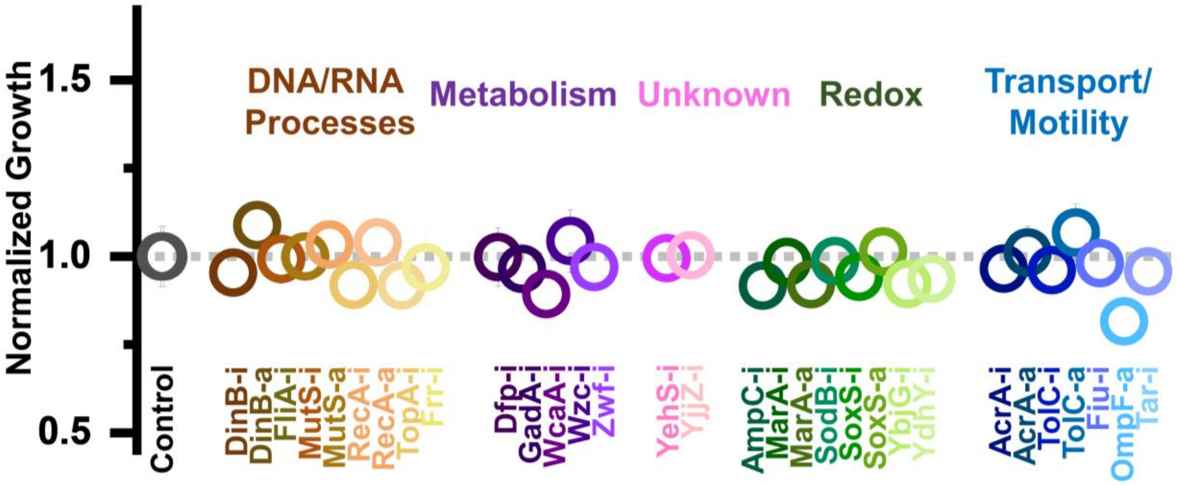

Supplementary Figure S1. Normalized growth of CRISPR perturbed strains in the absence of biofuel stress. Growth is normalized to the control strain.

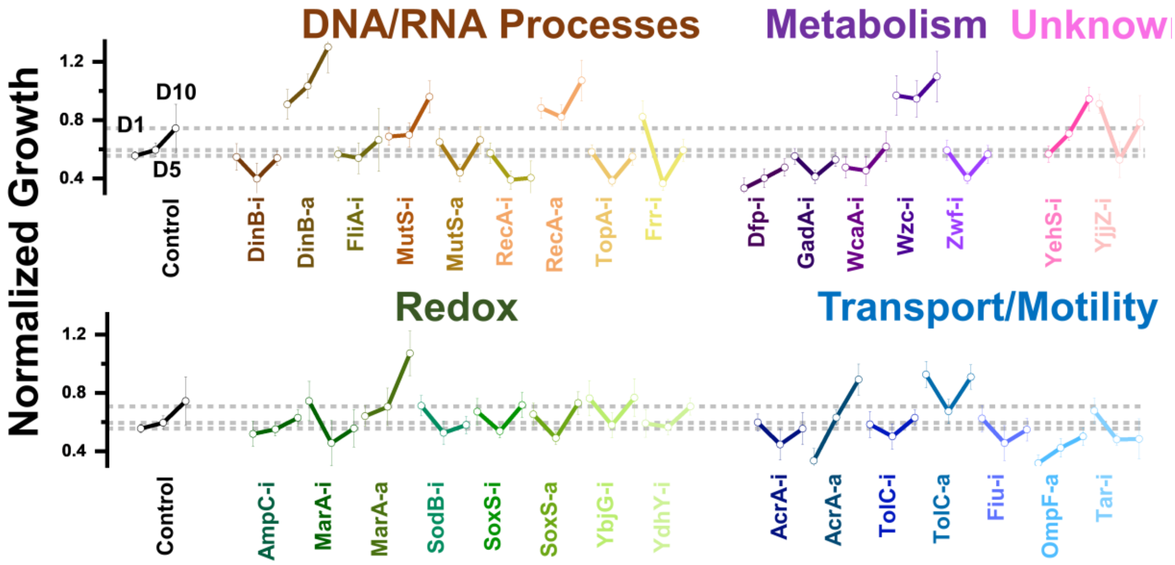

Supplementary Figure S2. Growth of CRISPR perturbed strains grown during exposure to 0.5% n-butanol, normalized to growth of each corresponding strain in the absence of biofuel stress. Error bars represent propagation of error from Figure 3A.

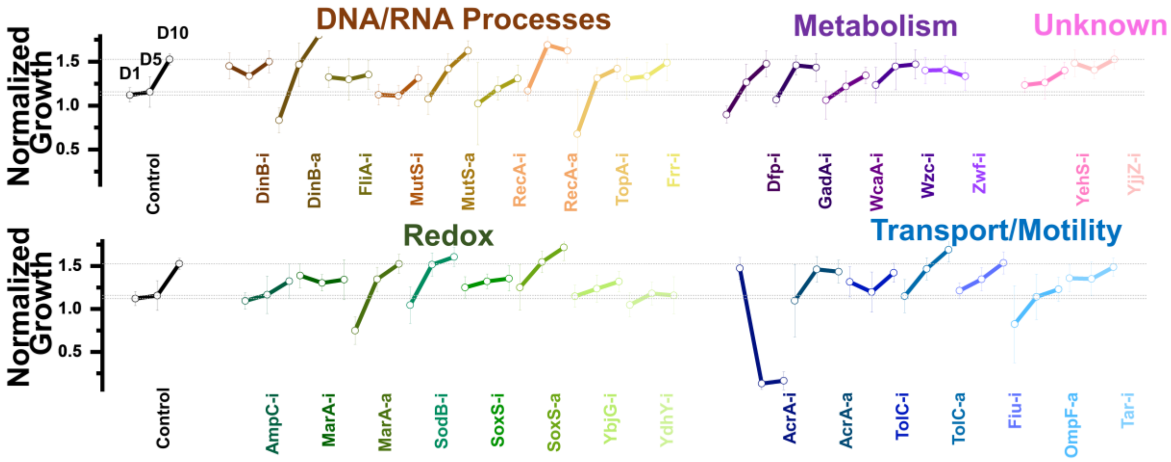

Supplementary Figure S3. Growth of CRISPR perturbed strains grown during exposure to 10% n-hexane, normalized to growth of each corresponding strain in the absence of biofuel stress. Error bars represent propagation of error of data from Figure 5A.

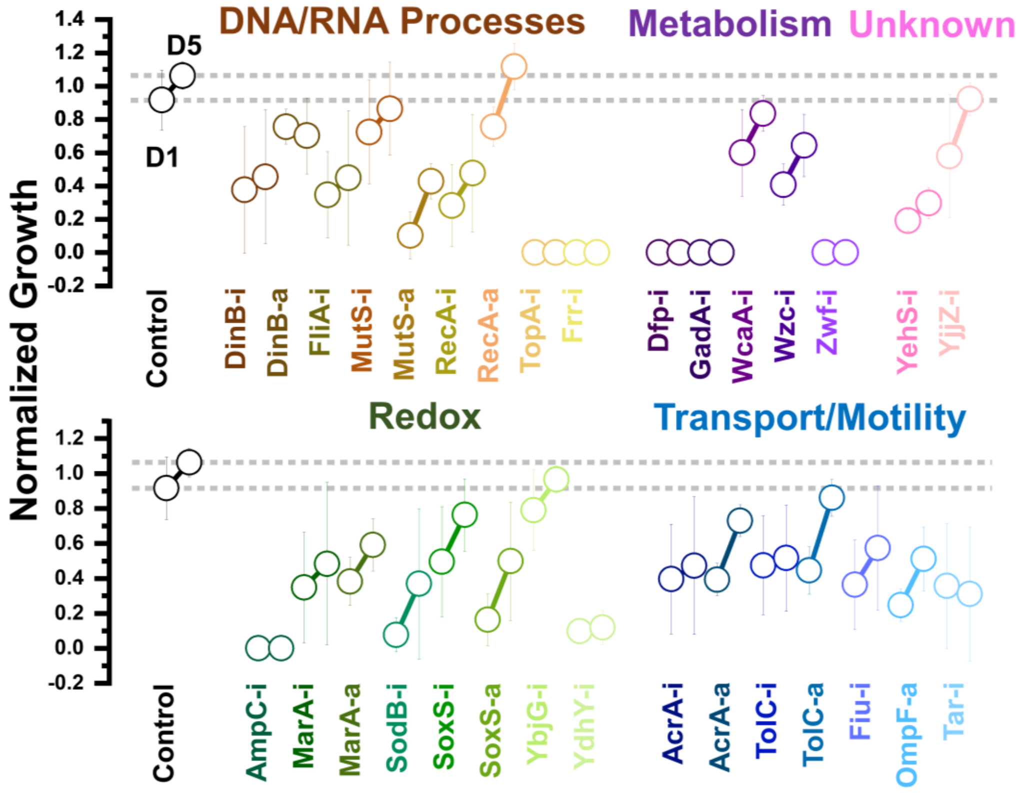

Supplementary Figure S4. Growth of hypermutator CRISPR perturbed strains grown during exposure to 1.0% n-butanol (top) or 10% n-hexane (bottom), normalized to growth of each corresponding strain in the absence of biofuel stress. Error bars represent propagation of error of data from Figure 7C.

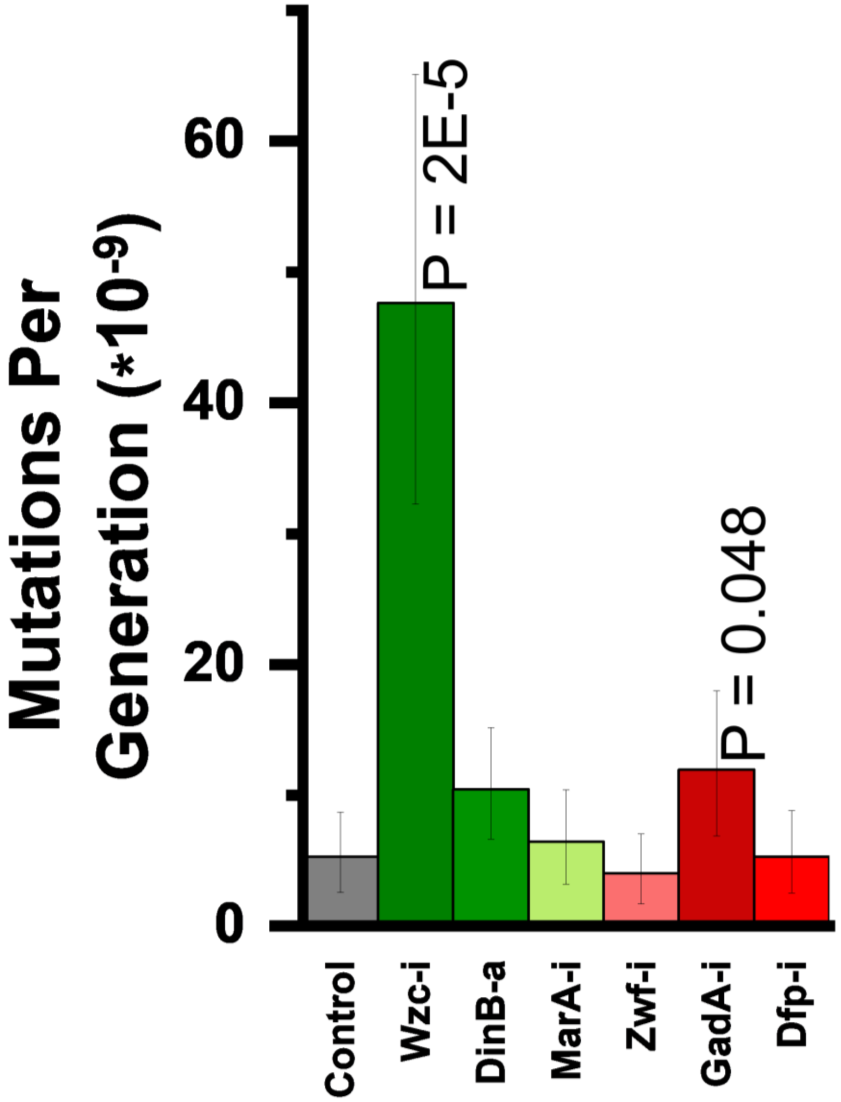

Supplementary Figure S5. Mutation rates of three strains growing well in n-butanol (green) and poorly in n-butanol (red). Data was collected as in Figure 7B, using 32 biological replicates. A two-tailed type II t-test was used to calculate statistical differences between strains, and those that exhibited significant differences ($P<0.05$) are labeled with their corresponding P-value. Error bars indicate 95% confidence interval.

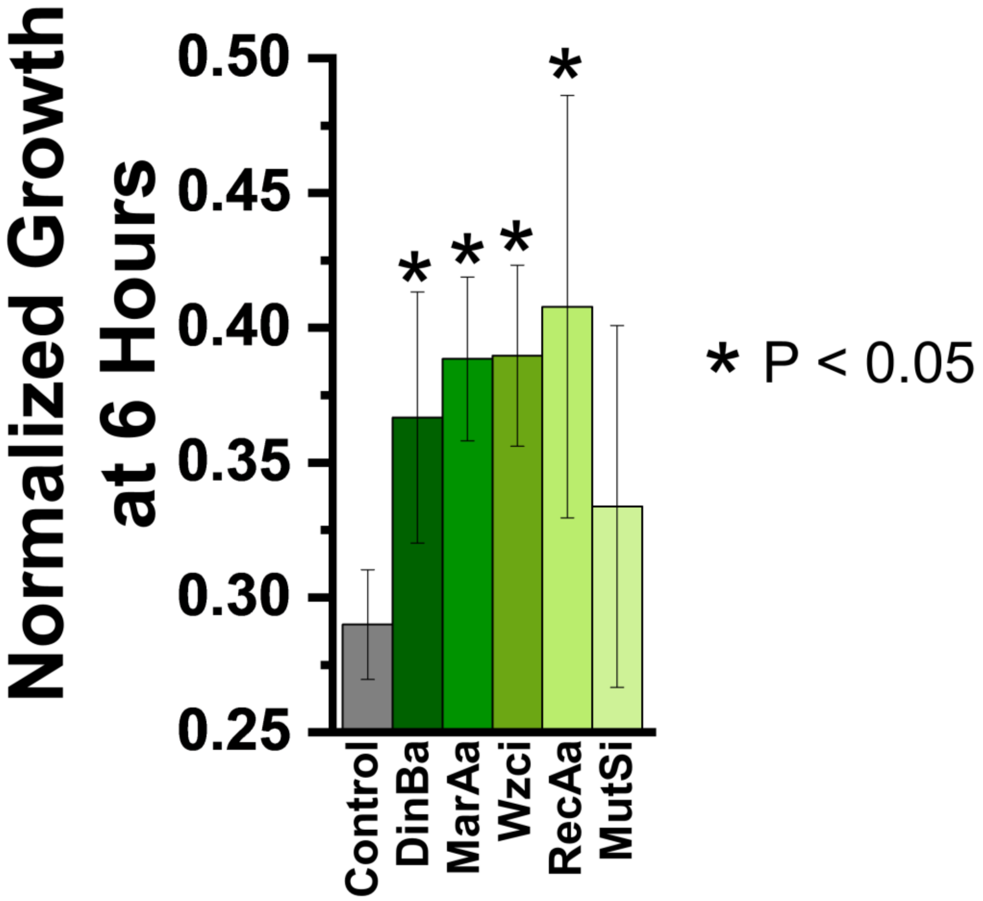

Supplementary Figure S6. Normalized growth of CRISPR perturbed strains grown in larger 15 mL batch culture volumes. The five strains growing to the highest concentrations after 10 days of exposure to 0.5% n-butanol were saved in glycerol stocks, and used to inoculate fresh 3 mL cultures of LB supplemented aTc and n-butanol. These cultures were grown overnight for 16 hours, and used to inoculate 15 mL batch cultures. ODs were measured at the beginning and end of six hours of growth and used to calculate normalized growth of each strain. All strains excluding MutS-i demonstrated sustained n-butanol tolerance in these larger culture volumes. Error bars represent standard deviations of four biological replicates. Significance was calculated using a two-tailed type two student's t-test in relation to the control RFP perturbed strain.

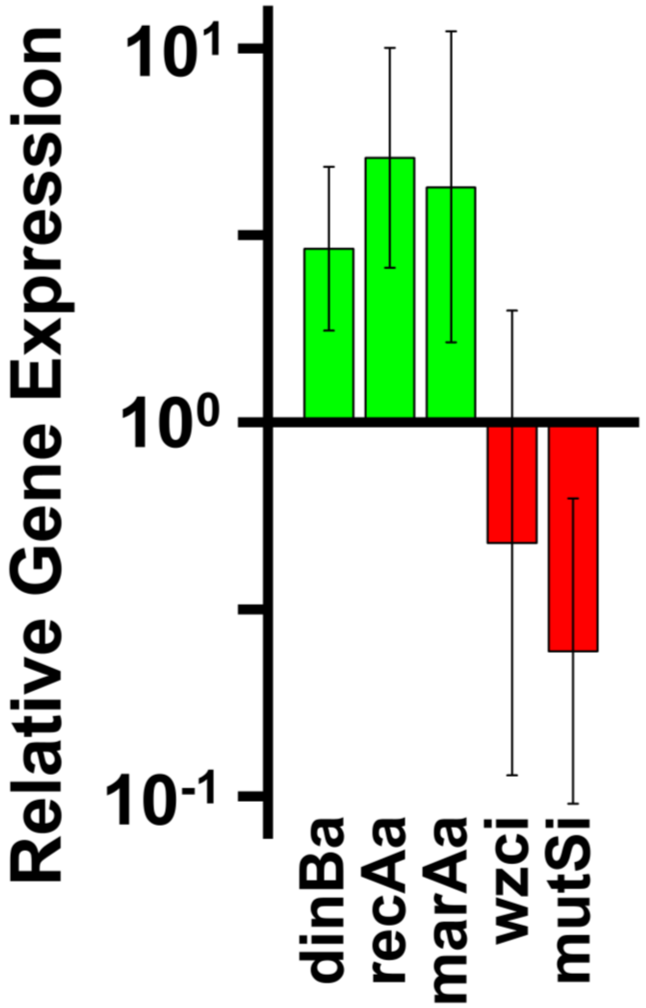

Supplementary Figure S7. CRISPR perturbation remains effective over prolonged periods of exposure. Samples form Supplementary Figure S2 were collected after 24 hours of growth and used for performing RT-qPCR. Error bars represent standard deviation of three biological replicates, using technical duplicates of each replicate during qPCR. The ΔΔCt method was used to calculate changes in gene expression caused by CRISPR perturbation in relation to the RFP perturbed control strain. All strains excluding inhibition of *wzc* demonstrated significant maintained perturbation of gene expression.

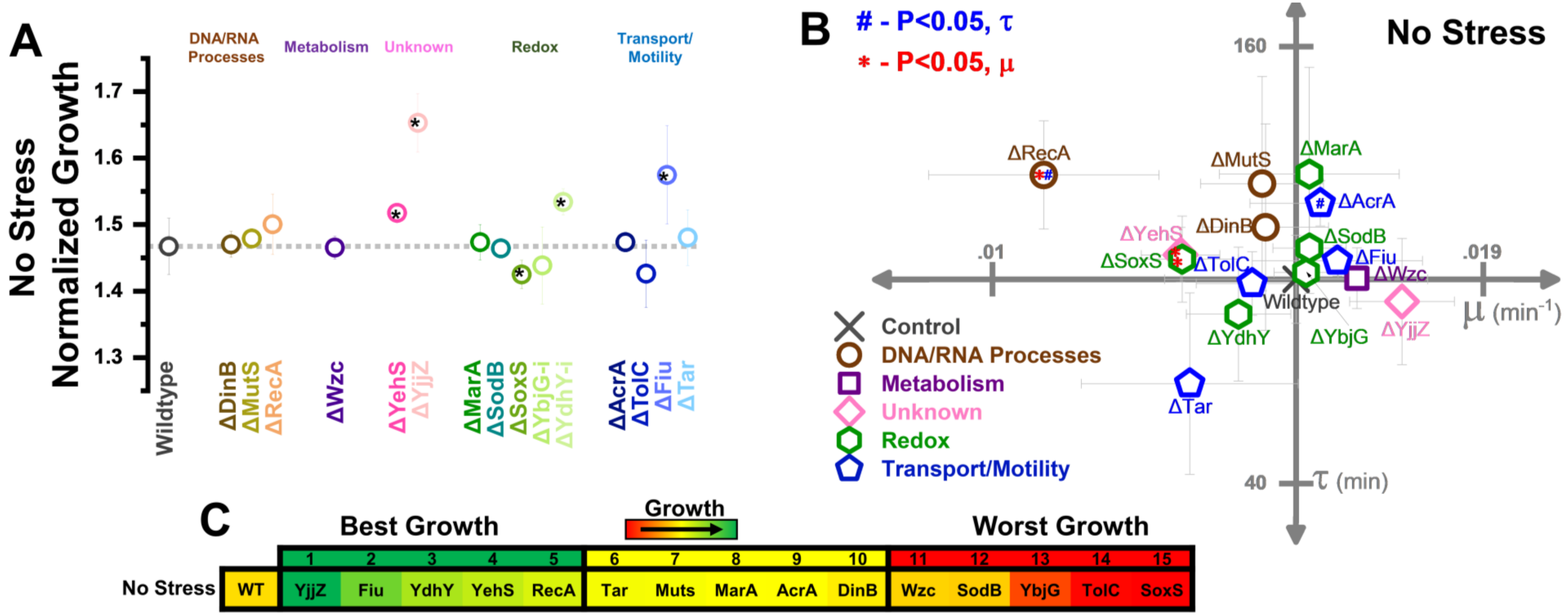

Supplementary Figure S8. Growth of gene knockouts in relation to wildtype *E. coli* BW25113 during exposure to no biofuel stress. (**A**) Normalized growth (maximum OD/starting OD) of knockouts. Strains are organized based on pathways affected by perturbation. Dashed lines extend from the control A two-tailed type II t-test was used to calculate significance (P<0.05) relative to the control. (**B**) Growth rates (μ) and lag times (τ) of knockouts during exposure to no biofuel stress. Axes are set to intersect the control in each graph. A two-tailed type II t-test was used to calculate significance (P<0.05) relative to the control in growth rates (red *) and lag times (blue #). (**C**) Organized rankings of strains with highest growth reached on each day, with the color scale to indicate relative growth. The top five and bottom five are indicated as "best growth" and "worst growth" respectively. All error bars represent the standard deviation of eight biological replicates

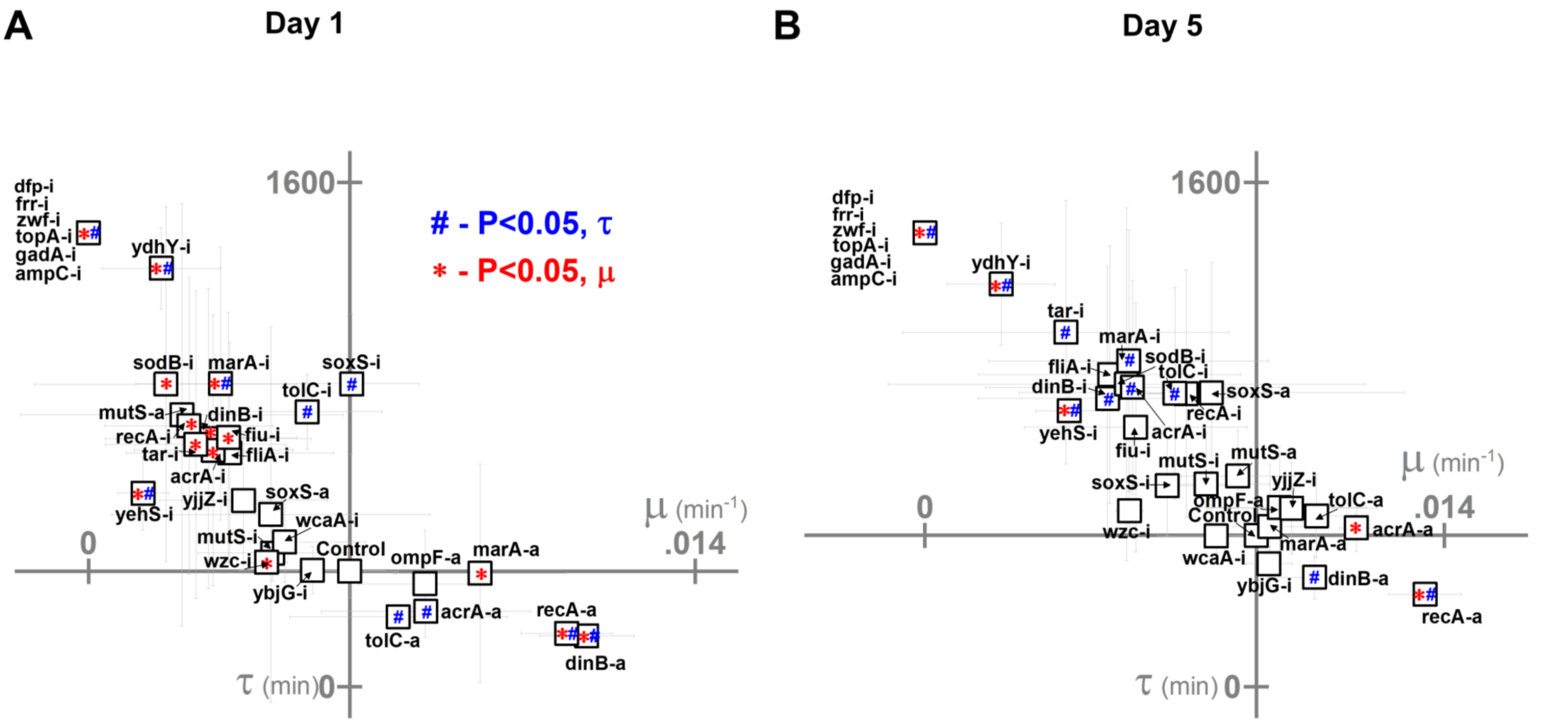

Supplementary Figure S9. Growth rates (μ) and lag times (τ) of hyper-mutator *E. coli* harboring CRISPR gene perturbations during 1.0% vol/vol n-butanol exposure. These growth characteristics were quantified on (**A**) day one and (**B**) and day five of the experiment. Scales are set to intersect the control in each graph. A two-tailed type II t-test was used to calculate significance ($P<0.05$) relative to the control in growth rates (red *) and lag times (blue #). Error bars represent the standard deviation of four biological replicates.

Table S1. The unique 20 nt sgRNA target used to activate or inhibit gene expression.

Gene Perturbation	Sequence
acrA-i	AGCATCAGAACGACCGCCAG
acrA-a	GAGCCACATCGAGGATGTGT
ampC-i	TAGGCGGGCCGGATTTACAT
dfp-i	GTGATAAAATCGCCAACTTC
dinB-i	ATTGTCGCGCATCTCCACTG
dinB-a	GCAAAAGCTGGATAAGCAGC
fiu-i	GTCCCTTTAACGCTAACAAA
fliA-i	TCGCTCACAAATAGGTAATG
frr-i	AGCCCTGATTAAACATATTA
gadA-i	AAAGTAGGATTTATCCGCAA
marA-i	CCAGTCCAAAATGCTATGAA
marA-a	GTTTTGTTCAATGCGATGCA
mutS-i	CTGCTGCATCATGGGCGTAT
mutS-a	GCAAGTACGCAAAATTGTAT
ompF-a	GTAACCAAAAGTAAAATTTA
recA-i	TACCAAATTGTTTCTCAATC
recA-a	CCGTGATGCGGTGCGTCGTC
sodB-i	ACCATATGCTAAAGATGCTC
soxS-i	CTACATCAATGTTAAGCGGC
soxS-a	GCGTTTCGCCACTTCGCCGG
tar-i	CGCGGATACGGTTAATCATA
tolC-i	GGCTCAGGCCGATAAGAATG
tolC-a	AGCAGTCATGTGTTAAATTG
topA-i	CTGGCAACGAGTTACCGATA
wcaA-i	TCTCAATCTATATGCCGACC
wzc-i	CAACATGCCGCTCCGGTAAC
ybjG-i	TATCTCTCTCTAAGTTTAAA
ydhY-i	GATCGTCCACTATTAGATAT
yehS-i	CGCACGCGATGTAAAACTTT
yjjZ-i	ATCATGTTGCAACGTACGCT
zwf-i	GTATACTTGTAATTTTCTTA
RFP (Control)	AACTTTCAGTTTAGCGGTCT

Table S2. Cloning primers used for transferring the error-prone Pol1 sequence into dCas9/ dCas9-ω plasmids.

Primer	Sequence
dCas9/ω Fwd	AATAACCTAGGAATCATGGCAATTCTGGAAGAAATAGCGC
dCas9/ω Rev	AATTTGACGTCTTACATGCTGTTCATCTGTTACATTGTCG
ePol1 Fwd	ACAGATGAACAGCATGTAAGACGTCAAATTTTAAGACCCACTTTCACATT
ePol1 Rev	TTCCAGAATTGCCATGATTCCTAGGTTATTTCTAGTAGAGAGCGTTCACC
